# Dataset on the folic acid uptake and the effect of sonication-based fortification on the color, pasting and textural properties of brown and milled rice

**DOI:** 10.1016/j.dib.2020.106198

**Published:** 2020-08-19

**Authors:** Rhowell N. Tiozon, Drexel H. Camacho, Aldrin P. Bonto, Glenn G. Oyong, Nese Sreenivasulu

**Affiliations:** a Chemistry Department, De La Salle University, 2401 Taft Avenue, Manila 0922 Philippines; b Organic Materials & Interfaces Unit, CENSER, De La Salle University, 2401 Taft Avenue, Manila 0922 Philippines; c Molecular Science Unit Laboratory, Center for Natural Science and Environmental Research, De La Salle University, 2401 Taft Avenue Manila Philippines; d Grain Quality and Nutrition Center, International Rice Research Institute, Los Baños, Laguna 4031 Philippines

**Keywords:** Folic acid, Rice, Fortification, Folate, Texture

## Abstract

The data included in this article are related to research paper entitled “Efficient fortification of folic acid in rice through ultrasonic treatment and absorption”. These datasets compile the folic acid uptake expressed in concentration and the effects of folic acid fortification on the physical properties of brown and milled rice. We reported the folic acid uptake of rice in increasing fortificant concentration through soaking, one-step, and stepwise fortification protocols. In addition, the data on the effects of fortification on the color, pasting, and textural properties of brown and milled rice were also presented.

Specifications table

**Table:** 

Subject	Chemistry
Specific subject area	Food fortification, texture
Type of data	Table, Graph, Figure
How data were acquired	Folates in rice were extracted using the tri-enzyme technique and were quantified by microbiological assay with *Lactobacillus rhamnosus* (ATCC 7469). Color of the rice was determined using CIE L*, a* and b* color space using videometer. While, pasting and textural properties were measured using rapid visco analyzer and TA.XT-Plus texture analyzer, respectively
Data format	Raw, Analyzed
Parameters for data collection	The sonicated fortified brown and milled rice were soaked in the range of folic acid concentration (i.e. 100-1000ppm). The changes in texture (i.e. hardness, adhesiveness, cohesiveness, and springiness) and color (i.e. L, a, b values) of fortified rice samples were measured. The pH value and RVA properties of the ground fortified and unfortified rice were measured.
Description of data collection	The maximum folic acid uptake of rice and optimum sonication-based fortification method were determined. The changes in physical parameters of sonicated-fortified rice such as color, pasting, and texture of fortified rice were determined.
Data source	Los Baños and Manila, Philippines
Data accessibility	The data are with this article.
Related research article	R.N. Tiozon Jr., D.H. Camacho, A.P. Bonto, G.G. Oyong, N. Sreenivasulu, Efficient fortification of folic acid in rice through ultrasonic treatment and absorption, Food Chem. 335 (2021) 127629. https://doi.org/10.1016/j.foodchem.2020.127629 [1].

Value of the data

• The data are useful as a guide when fortification of rice with folic acid becomes a public policy where large scale fortification is done to address folic acid deficiency among the poor.
• The data provided would be beneficial to government agencies usually mandated to develop fortified foods for social intervention to predict the level of fortification and to control the color, thermal, and textural properties to meet specific needs of different sectors of society.
• The comprehensive description on levels of fortification, changes in color, pasting, and textural properties of rice using two fortification protocols would be of benefit to researchers for comparison especially in dealing with other important nutrients.

## 1 Data Description

**Fig. 1:**
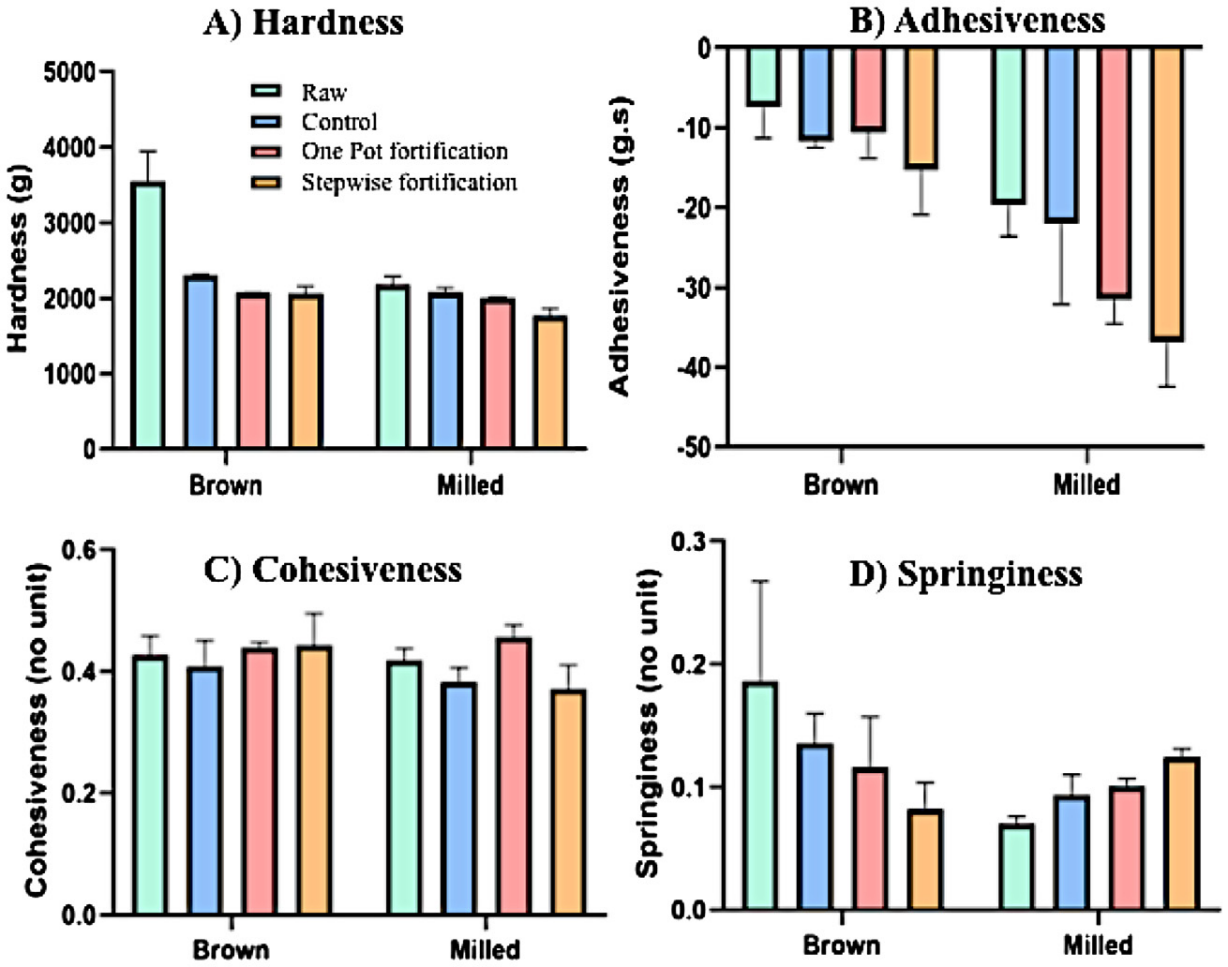
Texture profiles of unfortified and fortified samples

The data related to folic acid uptake in both the non-sonicated and sonicated rice upon soaking, one-pot, and stepwise fortifications were provided in Table 1, which includes the folic acid soaking concentration from 100 to 1000 ppm. The raw data for the folic acid concentrations, from which Table 1 was derived, are presented in the Supplementary Material 1 [S1]. Table 2 provides the data of color parameters L* (lightness), a* (red-green), and b* (yellow-blue) of rice samples were measured as 1976 CIE (*Commission International d'Eclairage*) L*, a* and b* color space. The fortified rice samples were evaluated against two references; non-sonicated unfortified (raw) rice samples and sonicated unfortified rice samples giving ΔL, Δa, Δb and ΔE_._ Moreover, ΔE_r_ and ΔE_s_ corresponds to the mean total color differences against raw (non-sonicated rice) and sonicated rice samples, respectively. The raw data for the colorimetric determination, from which Table 2 was derived, are presented in the Supplementary Material 2 [S2]. Data in Table 3 presents the pasting properties of raw, soaked, and fortified rice obtained using rapid viscoanalyzer. The raw data for the pasting properties, from which Table 3 was derived, are presented in the Supplementary Material 3 [S3]. Fig. 1 provides the instrumental textural properties (hardness, adhesiveness, cohesiveness and springiness) of unfortified and fortified rice samples. The raw data for the textural profiles, from which Fig. 1 was derived, are presented in the Supplementary Material 4 [S4].

**Table 1:** Concentration of Folic acid in rice in different fortification process and fortificant solution

	Folic acid content in fortified rice after soaking in different fortificant solution (x10^3^ μg/100g)									
Fortification method	100 ppm	200 ppm	300 ppm	400 ppm	500 ppm	600 ppm	700 ppm	800 ppm	900 ppm	1000 ppm
Soaking(brown)	6.75 ± 0.25	13.17 ± 0.98	16.05 ± 0.43	21.52 ± 1.22	29.59 ± 0.40	32.64 ± 0.27	33.00 ± 1.26	32.36 ± 0.84	32.78 ± 0.10	32.81 ± 0.14
Soaking (milled)	6.43 ± 0.25	14.91 ± 0.67	22.85 ± 0.57	31.88 ± 1.44	35.78 ± 1.75	36.41 ± 1.52	37.96 ± 1.13	41.11 ± 0.11	41.11 ± 0.11	41.15 ± 0.37
One-pot (brown)	6.93 ± 0.20	17.76 ± 0.67	23.47 ± 0.90	31.58 ± 1.23	31.97 ± 0.53	38.28 ± 0.76	39.98 ± 1.13	45.42 ± 0.06	45.42 ± 0.10	45.35 ± 0.33
One-pot (milled)	7.17 ± 0.10	17.91 ± 0.19	24.94 ± 0.09	33.48 ± 0.08	40.21 ± 0.03	45.23 ± 0.03	55.02 ± 2.95	61.03 ± 0.16	60.93 ± 0.15	61.03 ± 0.07
Stepwise (brown)	12.04 ± 0.11	18.64 ± 0.23	24.75 ± 0.61	32.22 ± 0.04	38.07 ± 0.11	42.26 ± 0.51	42.90 ± 2.95	51.95 ± 0.05	51.94 ± 0.06	52.01 ± 0.54
Stepwise (milled)	10.72 ± 0.11	23.88 ± 0.35	30.06 ± 0.02	36.45 ± 0.10	43.26 ± 0.76	50.93 ± 0.77	60.31 ± 0.55	65.59 ± 0.09	69.65 ± 0.08	69.70 ± 0.16

Data presented as mean ± standard deviation of triplicate determinations.

**Table 2:** Color parameters of rice samples

			Non-sonicated, unfortified(raw)	Sonicated, unfortified	Non-sonicated, fortified by soaking (control)	One-pot fortification	Stepwise fortification
Brown Rice	Color Parameters	L*	71.99 ± 2.59^a^	72.70 ± 3.07^a^	72.47 ± 2.60^a^	74.05 ± 2.29^a^	75.96 ± 2.40^a^
		a*	3.23 ± 0.88	3.26 ± 1.21	2.95 ± 1.00	2.52 ± 0.94	2.54 ± 0.80
		b*	17.06 ± 1.48^a^	16.91 ± 1.84^a^	17.57 ± 1.62^a^	18.06 ± 1.27^a^	21.83 ± 1.44^a^
	Difference with raw rice	Δ L		0.71	0.49	1.97	2.06
		Δ a		0.03	-0.28	-0.71	-0.69
		Δ b		-0.15 ^c^	0.52^a^	1.01^b^	4.77^abc^
		Δ E_r_		4.59^a^	3.62^a^	4.21^b^	6.29^ab^
	Difference with sonicated, unfortified	Δ L			1.26	1.26	1.35
		Δ a			-0.72	-0.74	-0.72
		Δ b			0.67^a^	1.16^b^	4.92^ab^
		Δ E_s_			4.27^a^	4.56^b^	6.57^ab^
Milled Rice	Color Parameters	L*	76.41 ± 1.23^a^	78.43 ± 1.09^ab^	79.41 ± 2.17^ac^	80.42 ± 2.20^abc^	84.06 ± 1.63^abc^
		a*	0.18 ± 0.10^a^	0.23 ± 0.09^b^	-1.40 ± 0.22^ab^	-1.21 ± 0.39^ab^	-1.07 ± 0.29^ab^
		b*	3.64 ± 0.61^a^	2.78 ± 0.46^b^	6.64 ± 1.35^ab^	7.20 ±1.88^ab^	10.43 ± 1.11^b^
	Difference with raw rice	Δ L		2.02^cd^	3.00^a^	4.01^bc^	7.65^abd^
		Δ a		-1.58^ab^	-1.39^c^	-1.25^ad^	0.05^bcd^
		Δ b		-0.86^ab^	3.01^a^	3.57^b^	6.79^ab^
		Δ E		5.04^ab^	5.93^cd^	10.42^ace^	2.60^bd^
	Difference with sonicated, unfortified	Δ L			0.98^a^	1.99^b^	5.63^ab^
		Δ a			-1.63^a^	-1.44	-1.30^a^
		Δ b			3.86^a^	4.42^b^	7.65^ab^
		Δ E			5.00^a^	5.66^b^	9.80^ab^

Data presented as mean ± standard deviation of triplicate determinations. Identical superscript letters indicate significant difference (P < 0.05). ΔE_r_ means total color difference against raw (non-sonicated rice) while ΔE_s_ means total color difference against sonicated rice samples.

**Table 3:** Pasting Properties of unfortified and fortified brown and milled rice samples.

	Brown Rice		Milled Rice	
	Unfortified	Fortified	Unfortified	Fortified
pH	6.67 ± 0.02^a^	6.60 ± 0.00^a^	6.36 ± 0.04^a^	5.94 ± 0.01^a^
Peak Viscosity (PV; cPa)	2235.00 ± 59.09^ab^	2726.67 ± 63.96^ab^	3811.33 ± 79.25^a^	3709.67 ± 45.35^b^
Trough Viscosity (TV; cPa)	1998.00 ± 27.73^ab^	2226.33 ± 52.00^ab^	2866.33 ± 29.02^a^	2898.67 ± 50.27^b^
Breakdown (BD; cPa)	237.00 ± 33.51^a^	500.33 ± 12.90^a^	945.00 ± 60.22^a^	811.00 ± 10.58^a^
Final Viscosity (FV; cPa)	3734.67 ± 56.89^a^	4311.67 ± 21.94^a^	5640.33 ± 137.93^a^	5324.33 ± 32.35^a^
Setback (SB; cPa)	1499.67 ± 53.97^a^	1585.00 ± 42.44^b^	1829.00 ± 69.40^abc^	1614.67 ± 30.50^c^
Pasting Temp (P_temp_;°C)	81.50 ± 1.39	79.90 ± 0.05	79.40 ± 0.44	76.73 ± 3.58
Retrogradation	1736.67 ± 55.14^a^	2085.33 ± 30.17^a^	2774.00 ± 126.41^a^	2425.67 ± 40.67^a^

Data presented as mean ± standard deviation of triplicate determinations. Values with identical superscript letters in the same row are significantly different (P < 0.05)

## 2 Experimental design, materials, and methods

### 2.1 Materials, Fortification, Extraction, and Quantification of Folic acid in Rice

Indica rice *IR54* were planted and grown during the dry season of 2017 under field conditions at IRRI following the standard agronomic practices. The paddy grains were harvested at maturity and the samples were then stored to equilibrate the moisture content to 14%. The samples were then dehulled (Zaccaria Testing Rice Mill Mod. PAZ-1/DTA, Limeira, Brazil) to obtain brown rice and further milled (Kett mill Grain Polisher Pearlest, Tokyo, Japan) for 45 s to obtain the milled rice samples. Then, 5.0 g of the brown and milled rice samples were ground to a fine powder (Mixer Mill MM 400, Retsch, Germany). The homogenized rice flour was used for folate analysis. Folic acid standard was obtained from Sigma-Aldrich (Singapore). Food grade Folic acid (lot No. UT16010029) was obtained from DSM (Heerlen, the Netherlands). Milli-Q water was used throughout the study.

The brown and milled rice kernels (5.0 g) with measured low folate contents were soaked in distilled water in 1:1 rice/water ratio and subjected to bath ultrasonication for 5 mins (Soner 206H, 53KHz180W ultrasonic power; Rocker Scientific Co. Taipei, Taiwan). Ice was added in the bath to maintain the temperature at <15°C. Two fortification processes were employed in this study – stepwise and one-pot. In stepwise fortification, the sonicated rice was air-dried at room temperature for 12 h followed by soaking in a folic acid solution of varying concentrations (from 100 to 1000 ppm) for 60 min [2]. In the one-pot fortification protocol, simultaneous sonication and fortification was done in one step. The processes were compared to the control method where the raw rice (non-sonicated) was simply soaked in folic acid solution. All the fortified samples were air-dried at room temperature under subdued light to achieve a moisture content of <14%. The folates and folic acid from the rice samples were extracted using the tri-enzyme technique that involves the use of α-amylase, protease, and conjugase [3]. The concentrations were quantified by microbiological assay with *Lactobacillus rhamnosus* (ATCC 7469) [4].

### 2.2 Color of Raw and Fortified Rice

Twenty (20) unbroken rice kernels from raw and fortified rice samples were placed evenly in a 90 mm Petri dish for color quantification using the VideometerLab 4 (https://videometer.com), which was calibrated with respect to the intensity and geometric parameters, followed by light set up. The multi-spectral images of each individual seed were captured at 19 different wavelengths from 365 to 970 nm, each with a resolution of 2056 × 2056 pixels. The color difference metrics as defined by the 1976 CIE (*Commission International d'Eclairage*) and the color-appearance attributes such as the a* (red-green), b* (yellow-blue) and L (lightness) were used accordingly [5].

### 2.3 Pasting Properties of Fortified Rice

The pasting properties of the rice samples (raw and fortified) were measured using a Rapid Visco Analyzer (RVA, Model 4-D, Newport Scientific, Warriewood, Australia). The rice samples were first ground to afford the flour and 3.0 g of the rice flour was suspended in reverse osmosis-purified (RO) water (25 g) in a canister. The changes in viscosity were determined in RVA using the process described in AACC Method 61–02 [6] using the following time/temperature profile: heat (50–95°C) – hold (95°C) – cool (95–50°C). The time/temperature profile was controlled, and the data was collected and processed using the Thermocline for Windows (TCW) version 2.6.

### 2.4 Textural Properties of Fortified Rice

The textural profile of rice samples was done by first submerging 25 unbroken rice grains in water in a test tube using the rice-to-water ratio of 1:2. The test tube was covered to minimize the evaporation of water and The test tube was heated in boiling water for 30 min and then placed in a water bath (50°C) until texture profile analysis. Three cooked unbroken grains were subjected to a two-cycle compression test using TA.XT-plus texture profile analyzer (TPA) equipped with a cylindrical probe having a diameter of 35 mm (Stable Micro Systems Ltd., Surrey, UK). The texture profile resulting from this two-compression test, also known as a two-bite test, is composed of two sets of curves (one positive and one negative), which can be divided into regions that represent down-strokes (increasing values) and up-strokes (decreasing values). Hardness represented the peak of the first positive curve. Adhesiveness was the area under the negative curve, representing the work required to pull the plunger from the sample on the base plate. The cohesiveness was the ratio of the area of the second positive curve to that of the first positive curve. Springiness, the ratio of the time elapsed from the upstroke to the peak in the second curve (T2) to the time elapsed from starting point to the peak of the first curve (T1), representing sample height recovery after the initial compression. These parameters were measured at 90% strain and test speed at 0.5 mm s^−1^. For each cooking replicate, three compression replicates were conducted [7].

## Ethics Statement

This work does not involve human or animal subjects.

CRediT author statement: Rhowell N. Tiozon Jr.: Conceptualization, Methodology, Investigation, Writing- Original draft preparation; Drexel H. Camacho: Conceptualization, Supervision, Writing- Reviewing and Editing; Aldrin P. Bonto: Conceptualization, Methodology, Investigation, Writing- Original draft preparation

Glenn G. Oyong: Methodology; Nese Sreenivasulu: Supervision, Writing- Reviewing and Editing

## Declaration of Competing Interest

The authors declare no known competing financial interests or personal relationships which have, or could be perceived to have, influenced the work reported in this article.

## Appendix Supplementary materials

Image, application 1
