# Supplementary material for: Dataset on the folic acid uptake and the effect of sonication-based fortification on the color, pasting and textural properties of brown and milled rice
Source: Data Brief. 2020 Aug 19;32:106198. doi: 10.1016/j.dib.2020.106198 (PMC7452647; doi:10.1016/j.dib.2020.106198)
Supplement: Supplementary file 1 [file mmc1.docx]

**Dataset on the folic acid uptake and the effect of sonication-based fortification on the**

**color, pasting and textural properties of brown and milled rice**

Rhowell N. Tiozon Jr.^1,4^, Drexel H. Camacho^1,2*^, Aldrin P. Bonto^1,4^, Glenn G. Oyong^3^,

Nese Sreenivasulu^4^

^1^Chemistry Department, De La Salle University, 2401 Taft Avenue, Manila 0922 Philippines

^2^Organic Materials & Interfaces Unit, CENSER, De La Salle University, 2401 Taft Avenue, Manila 0922 Philippines

^3^Molecular Science Unit Laboratory, Center for Natural Science and Environmental Research, De La Salle University, 2401 Taft Avenue Manila Philippines

^4^Grain Quality and Nutrition Center, International Rice Research Institute, Los Baños, Laguna 4031 Philippines

E-mails: Drexel H. Camacho ([drexel.camacho@dlsu.edu.ph](mailto:drexel.camacho@dlsu.edu.ph))

**SUPPLEMENTARY MATERIAL**

**Index of Tables**

| **Table Number** | **Title** | **Page** |
| --- | --- | --- |
| **Table S1** | Raw data for the folic acid concentrations, from which Table 1 was derived | **S2** |
| **Table S2** | Raw data for the colorimetric determination, from which Table 2 was derived | **S3** |
| **Table S3A** | Raw data for the pasting properties (Part 1), from which Table 3 was derived | **S4** |
| **Table S3B** | Raw data for the pasting properties (Part 2), from which Table 3 was derived | **S7** |
| **Table S4** | Raw data for the textural profiles, from which Figure 1 was derived | **S8** |

**Table S1** Raw data for the folic acid concentrations, from which Table 1 was derived

| **FOLIC ACID CONCENTRATION RAW DATA** | | | | | | | | | | | | | | | |
| --- | --- | --- | --- | --- | --- | --- | --- | --- | --- | --- | --- | --- | --- | --- | --- |
|  | **Folic acid content in fortified rice after soaking in different fortificant solution (x10^3 μg/100g)** | | | | | | | | | | | | | | |
| Fortification method | **100 ppm** | | | AVE | SD | **200 ppm** | | | AVE | SD | **300 ppm** | | | AVE | SD |
| Soaking (brown) | 6.62 | 6.87 | 6.76 | 6.75 | 0.12 | 12.90 | 13.73 | 12.86 | 13.16 | 0.49 | 16.26 | 16.06 | 15.83 | 16.05 | 0.22 |
| Soaking (milled) | 6.34 | 6.51 | 6.43 | 6.43 | 0.09 | 14.50 | 15.38 | 14.84 | 14.91 | 0.44 | 22.51 | 22.78 | 23.26 | 22.85 | 0.38 |
| One-pot (brown) | 6.87 | 7.04 | 6.87 | 6.93 | 0.10 | 17.71 | 18.11 | 17.45 | 17.76 | 0.33 | 23.22 | 23.19 | 23.99 | 23.47 | 0.45 |
| One-pot (milled) | 7.14 | 7.25 | 7.12 | 7.17 | 0.07 | 17.79 | 17.91 | 18.04 | 17.91 | 0.12 | 25.00 | 24.89 | 24.93 | 24.94 | 0.06 |
| Stepwise (brown) | 12.01 | 12.00 | 12.10 | 12.04 | 0.05 | 18.54 | 18.76 | 18.60 | 18.64 | 0.12 | 24.55 | 25.10 | 24.59 | 24.75 | 0.30 |
| Stepwise (milled) | 10.69 | 10.80 | 10.67 | 10.72 | 0.07 | 23.79 | 24.14 | 23.70 | 23.88 | 0.23 | 30.05 | 30.07 | 30.05 | 30.06 | 0.01 |
|  |  |  |  |  |  |  |  |  |  |  |  |  |  |  |  |
| Fortification method | **400 ppm** | | | AVE | SD | **500 ppm** | | | AVE | SD | **600 ppm** | | | AVE | SD |
| Soaking (brown) | 21.82 | 21.92 | 20.81 | 21.52 | 0.61 | 29.82 | 29.47 | 29.48 | 29.59 | 0.20 | 32.70 | 32.73 | 32.48 | 32.63 | 0.13 |
| Soaking (milled) | 32.06 | 32.73 | 30.84 | 31.88 | 0.96 | 36.86 | 35.92 | 34.55 | 35.78 | 1.16 | 37.50 | 35.50 | 36.22 | 36.41 | 1.01 |
| One-pot (brown) | 30.99 | 32.21 | 31.48 | 31.56 | 0.62 | 31.66 | 32.12 | 32.12 | 31.96 | 0.26 | 37.90 | 38.66 | 38.28 | 38.28 | 0.38 |
| One-pot (milled) | 33.46 | 33.54 | 33.45 | 33.48 | 0.05 | 40.29 | 40.30 | 40.04 | 40.21 | 0.14 | 45.23 | 45.25 | 45.21 | 45.23 | 0.02 |
| Stepwise (brown) | 32.21 | 32.21 | 32.24 | 32.22 | 0.02 | 38.02 | 38.08 | 38.12 | 38.07 | 0.05 | 42.01 | 42.53 | 42.24 | 42.26 | 0.26 |
| Stepwise (milled) | 36.43 | 36.39 | 36.52 | 36.45 | 0.07 | 42.94 | 42.99 | 43.84 | 43.26 | 0.50 | 50.52 | 50.77 | 51.51 | 50.93 | 0.52 |
|  |  |  |  |  |  |  |  |  |  |  |  |  |  |  |  |
| Fortification method | **700 ppm** | | | AVE | SD | **800 ppm** | | | AVE | SD | **900 ppm** | | | AVE | SD |
| Soaking (brown) | 32.82 | 33.70 | 32.48 | 33.00 | 0.63 | 32.84 | 32.12 | 32.11 | 32.35 | 0.42 | 32.82 | 32.73 | 32.79 | 32.78 | 0.05 |
| Soaking (milled) | 38.39 | 38.39 | 37.10 | 37.96 | 0.75 | 38.40 | 38.40 | 37.10 | 37.97 | 0.75 | 41.15 | 41.15 | 41.03 | 41.11 | 0.07 |
| One-pot (brown) | 39.42 | 40.55 | 39.97 | 39.98 | 0.56 | 45.41 | 45.39 | 45.44 | 45.41 | 0.03 | 45.44 | 45.36 | 45.44 | 45.41 | 0.05 |
| One-pot (milled) | 56.14 | 56.17 | 52.75 | 55.02 | 1.96 | 60.96 | 60.96 | 61.15 | 61.03 | 0.11 | 61.00 | 60.96 | 60.82 | 60.93 | 0.10 |
| Stepwise (brown) | 42.87 | 42.95 | 42.87 | 42.89 | 0.05 | 51.95 | 51.93 | 51.98 | 51.95 | 0.03 | 51.93 | 51.98 | 51.93 | 51.94 | 0.03 |
| Stepwise (milled) | 60.10 | 60.10 | 60.74 | 60.31 | 0.37 | 69.56 | 69.56 | 69.66 | 69.59 | 0.06 | 69.59 | 69.70 | 69.66 | 69.65 | 0.05 |
|  |  |  |  |  |  |  |  |  |  |  |  |  |  |  |  |
| Fortification method | **1000 ppm** | | | AVE | SD |  |  |  |  |  |  |  |  |  |  |
| Soaking (brown) | 32.85 | 32.73 | 32.85 | 32.81 | 0.07 |  |  |  |  |  |  |  |  |  |  |
| Soaking (milled) | 41.27 | 41.03 | 41.15 | 41.15 | 0.12 |  |  |  |  |  |  |  |  |  |  |
| One-pot (brown) | 45.44 | 45.16 | 45.44 | 45.35 | 0.16 |  |  |  |  |  |  |  |  |  |  |
| One-pot (milled) | 61.00 | 61.08 | 61.00 | 61.03 | 0.04 |  |  |  |  |  |  |  |  |  |  |
| Stepwise (brown) | 51.98 | 51.76 | 52.30 | 52.01 | 0.27 |  |  |  |  |  |  |  |  |  |  |
| Stepwise (milled) | 69.80 | 69.59 | 69.70 | 69.70 | 0.10 |  |  |  |  |  |  |  |  |  |  |

**Table S2** Raw data for the colorimetric determination, from which Table 2 was derived

| **S2: COLORIMETER RAW DATA** | | | | | | | | | | | | | | | | | | |
| --- | --- | --- | --- | --- | --- | --- | --- | --- | --- | --- | --- | --- | --- | --- | --- | --- | --- | --- |
| **Non-sonicated, unfortified (raw)** | | |  | **Sonicated, unfortified** | | |  | **Non-sonicated, fortified by soaking (control)** | | |  | **One-pot fortification** | | |  | **Stepwise fortification** | | |
| **L*** | **a*** | **b*** |  | **L*** | **a*** | **b*** |  | **L*** | **a*** | **b*** |  | **L*** | **a*** | **b*** |  | **L*** | **a*** | **b*** |
| 71.3960 | 2.7571 | 15.1118 |  | 72.1047 | 2.5482 | 15.5818 |  | 74.4033 | 2.0924 | 17.5017 |  | 74.3850 | 2.8300 | 19.3583 |  | 74.0097 | 1.9503 | 21.2654 |
| 74.6073 | 2.2711 | 13.8507 |  | 75.9602 | 2.6512 | 16.7484 |  | 70.1255 | 3.0520 | 19.7118 |  | 72.1999 | 3.2273 | 18.1621 |  | 75.7777 | 4.0883 | 21.2867 |
| 72.0304 | 3.2788 | 17.8890 |  | 75.3312 | 1.3856 | 12.8718 |  | 73.0091 | 2.9467 | 16.0714 |  | 75.7267 | 1.7534 | 17.7840 |  | 72.2407 | 2.8404 | 20.1066 |
| 72.6202 | 2.1723 | 16.8062 |  | 68.1891 | 4.7607 | 18.6186 |  | 74.5570 | 3.4770 | 17.1793 |  | 73.9645 | 1.8433 | 18.2550 |  | 75.4808 | 3.6163 | 22.0032 |
| 70.1132 | 2.5934 | 16.3807 |  | 78.8460 | 1.9965 | 14.9675 |  | 74.0608 | 2.0174 | 17.1545 |  | 75.4041 | 1.8846 | 18.1648 |  | 78.0317 | 1.7430 | 20.1881 |
| 69.8872 | 4.1715 | 18.2752 |  | 73.5705 | 2.6886 | 16.7052 |  | 69.6312 | 5.2059 | 17.3702 |  | 70.7575 | 4.1235 | 20.1084 |  | 77.5412 | 1.8077 | 23.0128 |
| 68.6295 | 4.2955 | 18.3060 |  | 75.3972 | 1.8898 | 15.0382 |  | 69.8902 | 3.4412 | 17.7988 |  | 69.7614 | 3.8008 | 18.4694 |  | 75.3537 | 3.3880 | 24.3014 |
| 70.4883 | 4.1287 | 18.3586 |  | 71.2822 | 3.6214 | 17.0878 |  | 66.9411 | 4.4770 | 16.6511 |  | 75.5382 | 1.2027 | 14.6815 |  | 74.3352 | 2.5517 | 21.9756 |
| 76.3169 | 2.5052 | 16.3276 |  | 77.2574 | 1.7529 | 14.6367 |  | 72.9003 | 2.8215 | 17.8764 |  | 77.0201 | 1.8913 | 16.4338 |  | 75.4548 | 1.9761 | 23.1775 |
| 72.6244 | 2.6684 | 16.4061 |  | 71.9681 | 3.5406 | 17.0827 |  | 73.2918 | 1.6680 | 17.4281 |  | 70.4749 | 3.3137 | 18.4169 |  | 80.0840 | 1.5427 | 18.2101 |
| 70.1239 | 4.3479 | 18.8917 |  | 73.1355 | 2.9631 | 15.8976 |  | 70.2021 | 3.8653 | 18.9993 |  | 73.5353 | 2.9842 | 17.9544 |  | 73.7318 | 3.1525 | 23.9311 |
| 78.0342 | 2.0535 | 15.5522 |  | 68.4567 | 4.9304 | 18.3892 |  | 75.0300 | 1.9195 | 17.0128 |  | 73.7556 | 2.5031 | 17.6842 |  | 75.9128 | 1.5345 | 21.8246 |
| 70.0578 | 3.9674 | 19.0498 |  | 70.3311 | 3.8629 | 17.2197 |  | 72.3663 | 3.0532 | 17.5219 |  | 75.5487 | 3.3413 | 19.3138 |  | 75.7473 | 2.0328 | 21.1438 |
| 68.8988 | 4.3694 | 18.1847 |  | 70.6694 | 4.8517 | 20.6744 |  | 76.3840 | 2.2706 | 16.4380 |  | 76.5543 | 1.7357 | 17.9711 |  | 75.8596 | 2.2648 | 22.2397 |
| 70.7479 | 3.0349 | 16.5243 |  | 75.0140 | 2.9632 | 17.9197 |  | 72.2498 | 2.7424 | 17.7437 |  | 71.5980 | 3.6324 | 19.6262 |  | 75.3801 | 2.7686 | 23.8431 |
| 72.4579 | 3.7882 | 17.7030 |  | 69.2770 | 5.4274 | 18.4438 |  | 69.8770 | 3.2877 | 16.1712 |  | 72.6513 | 2.2457 | 17.7755 |  | 80.2240 | 3.3378 | 22.3070 |
| 70.1767 | 3.6827 | 18.0447 |  | 73.5274 | 2.2434 | 16.1667 |  | 72.8967 | 3.6144 | 17.2196 |  | 78.1015 | 0.8840 | 16.3701 |  | 73.4731 | 2.7699 | 21.1398 |
| 73.3175 | 2.3333 | 16.0862 |  | 70.9303 | 4.0900 | 18.5608 |  | 76.4763 | 1.9139 | 18.2032 |  | 72.9444 | 3.1332 | 18.5002 |  | 78.7622 | 1.8225 | 21.7432 |
| 70.9329 | 4.1855 | 18.5264 |  | 74.4851 | 2.4176 | 16.1945 |  | 75.4072 | 1.3041 | 17.5709 |  | 75.2535 | 1.2319 | 16.9355 |  | 75.5325 | 3.7610 | 21.0432 |
| 76.2486 | 1.9958 | 14.8340 |  | 68.2462 | 4.6842 | 19.3327 |  | 69.7772 | 3.9055 | 19.9399 |  | 75.7530 | 2.8479 | 19.2836 |  | 76.2180 | 1.8862 | 21.7773 |

**Table S3A** Raw data for the pasting properties (Part 1), from which Table 3 was derived

| **S3A: PASTING PROPERTIES RAW DATA PART 1** | | | | | | | | | | | | | | | | | | | | | | | | | |
| --- | --- | --- | --- | --- | --- | --- | --- | --- | --- | --- | --- | --- | --- | --- | --- | --- | --- | --- | --- | --- | --- | --- | --- | --- | --- |
| **2019-15** | | **BROWN_UNF T1** | | **BROWN_UNF T2** | | **BROWN_UNF T3** | | **BROWN_FORT 1** | | **BROWN_FORT 2** | | **BROWN_FORT 3** | | **MILLED_UNF 1** | | **MILLED_UNF 2** | | **MILLED_UNF 3** | | **MILLED_FORT 1** | | **MILLED_FORT 2** | | **MILLED_FORT 3** | |
| time (sec) | time (min) | visc (cP) | temp (°C) | visc (cP) | temp (°C) | visc (cP) | temp (°C) | visc (cP) | temp (°C) | visc (cP) | temp (°C) | visc (cP) | temp (°C) | visc (cP) | temp (°C) | visc (cP) | temp (°C) | visc (cP) | temp (°C) | visc (cP) | temp (°C) | visc (cP) | temp (°C) | visc (cP) | temp (°C) |
| 3.977778 | 0.0663 | 130 | 48.2 | 127 | 47.85 | 131 | 48.1 | 125 | 48.15 | 121 | 47.8 | 122 | 48.25 | 122 | 48.1 | 121 | 47.4 | 121 | 48 | 121 | 48.05 | 120 | 48.05 | 120 | 48.2 |
| 7.977778 | 0.1330 | 91 | 48 | 88 | 47.85 | 91 | 48.1 | 86 | 48.1 | 83 | 47.9 | 81 | 48.1 | 81 | 48.1 | 81 | 47.55 | 81 | 48.1 | 80 | 48 | 79 | 48 | 81 | 48.15 |
| 11.97778 | 0.1996 | 256 | 48.8 | 238 | 48.65 | 246 | 48.75 | 233 | 48.75 | 215 | 48.65 | 210 | 48.8 | 212 | 48.7 | 212 | 48.7 | 212 | 48.75 | 214 | 48.8 | 204 | 48.75 | 210 | 48.75 |
| 15.97778 | 0.2663 | 64 | 49.7 | 39 | 49.9 | 42 | 49.75 | 27 | 49.7 | 12 | 49.9 | 2 | 49.7 | 7 | 49.8 | 5 | 49.95 | 10 | 49.8 | 5 | 49.8 | -5 | 49.7 | -5 | 49.8 |
| 19.97778 | 0.3330 | 66 | 50.4 | 37 | 50.65 | 39 | 50.45 | 27 | 50.45 | 9 | 50.6 | 3 | 50.4 | 5 | 50.45 | 3 | 50.6 | 6 | 50.45 | 9 | 50.45 | -6 | 50.4 | -2 | 50.35 |
| 23.97778 | 0.3996 | 69 | 50.7 | 42 | 50.9 | 38 | 50.7 | 28 | 50.65 | 12 | 50.9 | 3 | 50.75 | 3 | 50.75 | 2 | 50.85 | 7 | 50.75 | 7 | 50.8 | -6 | 50.8 | 0 | 50.7 |
| 27.97778 | 0.4663 | 63 | 50.8 | 40 | 51 | 34 | 50.85 | 25 | 50.8 | 10 | 51 | 6 | 50.8 | 3 | 50.8 | 0 | 50.9 | 7 | 50.95 | 7 | 50.85 | -7 | 50.85 | -2 | 50.8 |
| 31.97778 | 0.5330 | 65 | 50.75 | 41 | 50.8 | 34 | 50.8 | 24 | 50.8 | 9 | 50.9 | 0 | 50.8 | 4 | 50.85 | 0 | 50.8 | 5 | 50.85 | 7 | 50.8 | -7 | 50.85 | -2 | 50.8 |
| 35.97778 | 0.5996 | 64 | 50.65 | 41 | 50.6 | 32 | 50.6 | 23 | 50.65 | 5 | 50.65 | -2 | 50.65 | 5 | 50.6 | -2 | 50.5 | 6 | 50.65 | 5 | 50.8 | -9 | 50.65 | -5 | 50.7 |
| 39.97778 | 0.6663 | 63 | 50.55 | 39 | 50.45 | 29 | 50.45 | 28 | 50.5 | 5 | 50.4 | -2 | 50.45 | 3 | 50.45 | -2 | 50.4 | 2 | 50.45 | 1 | 50.45 | -8 | 50.4 | -5 | 50.4 |
| 43.97778 | 0.7330 | 63 | 50.4 | 38 | 50.35 | 30 | 50.35 | 27 | 50.25 | 5 | 50.35 | -2 | 50.35 | 3 | 50.3 | -2 | 50.4 | 4 | 50.35 | 3 | 50.3 | -9 | 50.25 | -3 | 50.3 |
| 47.97778 | 0.7996 | 63 | 50.35 | 37 | 50.25 | 32 | 50.3 | 21 | 50.25 | 5 | 50.3 | 1 | 50.3 | 0 | 50.25 | -5 | 50.25 | 3 | 50.2 | 4 | 50.2 | -8 | 50.2 | -2 | 50.25 |
| 51.97778 | 0.8663 | 61 | 50.15 | 36 | 50.25 | 29 | 50.35 | 17 | 50.25 | 8 | 50.3 | -1 | 50.3 | 2 | 50.25 | -4 | 50.3 | 4 | 50.2 | 3 | 50.3 | -5 | 50.25 | -7 | 50.25 |
| 55.97778 | 0.9330 | 61 | 50.15 | 35 | 50.3 | 28 | 50.25 | 20 | 50.25 | 3 | 50.2 | -6 | 50.2 | 4 | 50.2 | -6 | 50.25 | 2 | 50.25 | 3 | 50.25 | -7 | 50.25 | -5 | 50.2 |
| 59.97778 | 0.9996 | 60 | 50.15 | 29 | 50.25 | 34 | 50.15 | 24 | 50.2 | 7 | 50.25 | -4 | 50.15 | -1 | 50.2 | -4 | 50.15 | 1 | 50.3 | 1 | 50.2 | -6 | 50.2 | -8 | 50.35 |
| 63.97778 | 1.0663 | 57 | 50.2 | 32 | 50.05 | 29 | 50.15 | 16 | 50.2 | 5 | 50.25 | -5 | 50.2 | 1 | 50.2 | -8 | 50.15 | 0 | 50.15 | 1 | 50.15 | -7 | 50.15 | -9 | 50.2 |
| 67.97778 | 1.1330 | 56 | 50.35 | 29 | 50.3 | 31 | 50.35 | 18 | 50.4 | 4 | 50.35 | -6 | 50.35 | 0 | 50.35 | -7 | 50.4 | -1 | 50.25 | 2 | 50.25 | -9 | 50.4 | -10 | 50.4 |
| 71.97778 | 1.1996 | 58 | 50.9 | 32 | 50.9 | 34 | 50.9 | 19 | 50.95 | 2 | 50.9 | -7 | 50.9 | 1 | 50.95 | -9 | 50.85 | 0 | 50.85 | 0 | 50.85 | -7 | 50.8 | -7 | 50.85 |
| 75.97778 | 1.2663 | 56 | 51.7 | 31 | 51.65 | 32 | 51.55 | 17 | 51.65 | 3 | 51.6 | -5 | 51.8 | -2 | 51.65 | -3 | 51.65 | 0 | 51.55 | 0 | 51.75 | -11 | 51.8 | -7 | 51.65 |
| 79.97778 | 1.3330 | 56 | 52.5 | 27 | 52.6 | 30 | 52.6 | 15 | 52.7 | 4 | 52.55 | -7 | 52.65 | -1 | 52.65 | -4 | 52.65 | -1 | 52.55 | 0 | 52.55 | -6 | 52.55 | -9 | 52.6 |
| 83.97778 | 1.3996 | 56 | 53.55 | 28 | 53.65 | 30 | 53.6 | 15 | 53.65 | 4 | 53.6 | -12 | 53.6 | -1 | 53.65 | -6 | 53.55 | 0 | 53.65 | 0 | 53.6 | -9 | 53.55 | -8 | 53.6 |
| 87.97778 | 1.4663 | 55 | 54.6 | 28 | 54.5 | 28 | 54.6 | 14 | 54.55 | 0 | 54.5 | -5 | 54.55 | 4 | 54.5 | -14 | 54.65 | -2 | 54.6 | -3 | 54.7 | -9 | 54.65 | -9 | 54.65 |
| 91.97778 | 1.5330 | 54 | 55.55 | 29 | 55.6 | 25 | 55.65 | 14 | 55.7 | 2 | 55.55 | -7 | 55.6 | -3 | 55.65 | -5 | 55.5 | 0 | 55.6 | -2 | 55.55 | -8 | 55.55 | -12 | 55.6 |
| 95.97778 | 1.5996 | 54 | 56.5 | 26 | 56.6 | 26 | 56.65 | 16 | 56.5 | 3 | 56.55 | -11 | 56.65 | -2 | 56.45 | -3 | 56.55 | 3 | 56.7 | -2 | 56.55 | -7 | 56.55 | -10 | 56.65 |
| 99.97778 | 1.6663 | 55 | 57.45 | 27 | 57.35 | 28 | 57.4 | 10 | 57.45 | 1 | 57.45 | -6 | 57.45 | -4 | 57.45 | -3 | 57.55 | 0 | 57.45 | -1 | 57.4 | -10 | 57.5 | -14 | 57.35 |
| 103.9778 | 1.7330 | 52 | 58.3 | 25 | 58.35 | 25 | 58.3 | 15 | 58.35 | 1 | 58.4 | -9 | 58.35 | -3 | 58.4 | -7 | 58.35 | 2 | 58.35 | -4 | 58.45 | -14 | 58.25 | -11 | 58.25 |
| 107.9778 | 1.7996 | 52 | 59.2 | 27 | 59.1 | 26 | 59.1 | 15 | 59.15 | 2 | 59.2 | -12 | 59.3 | 1 | 59.1 | -10 | 59.2 | 1 | 59.15 | -2 | 59.2 | -7 | 59.25 | -9 | 59.05 |
| 111.9778 | 1.8663 | 52 | 60 | 30 | 60.05 | 30 | 60.1 | 11 | 60.1 | 4 | 60.1 | -7 | 60.05 | -2 | 60.1 | -3 | 60.15 | 3 | 60.1 | 0 | 60.05 | -7 | 60.1 | -13 | 60.05 |
| 115.9778 | 1.9330 | 55 | 60.9 | 27 | 60.95 | 25 | 60.95 | 12 | 60.95 | 0 | 61 | -5 | 60.9 | -2 | 60.95 | -8 | 60.8 | 0 | 60.95 | 2 | 60.9 | -7 | 60.95 | -9 | 60.95 |
| 119.9778 | 1.9996 | 52 | 61.85 | 20 | 61.65 | 29 | 61.65 | 13 | 61.75 | 3 | 61.7 | -7 | 61.65 | -6 | 61.65 | -4 | 61.75 | 0 | 61.7 | -3 | 61.75 | -12 | 61.7 | -11 | 61.7 |
| 123.9778 | 2.0663 | 50 | 62.45 | 27 | 62.5 | 27 | 62.45 | 15 | 62.55 | 0 | 62.55 | -8 | 62.45 | -3 | 62.5 | -5 | 62.55 | 0 | 62.5 | -2 | 62.5 | -14 | 62.45 | -14 | 62.55 |
| 127.9778 | 2.1330 | 48 | 63.35 | 22 | 63.4 | 26 | 63.4 | 12 | 63.2 | 2 | 63.4 | -6 | 63.3 | -5 | 63.25 | -4 | 63.35 | -2 | 63.4 | -2 | 63.25 | -9 | 63.4 | -9 | 63.35 |
| 131.9778 | 2.1996 | 49 | 64.05 | 24 | 64.1 | 29 | 64.05 | 15 | 64.1 | -2 | 64.1 | -12 | 64.1 | -2 | 64.1 | -8 | 64.15 | -2 | 64.1 | -2 | 64 | -11 | 64.2 | -10 | 64.15 |
| 135.9778 | 2.2663 | 52 | 64.85 | 27 | 64.85 | 27 | 64.95 | 13 | 64.95 | -2 | 64.9 | -9 | 64.95 | -5 | 64.95 | -8 | 64.8 | 1 | 65 | 0 | 64.9 | -12 | 64.9 | -11 | 65 |
| 139.9778 | 2.3330 | 48 | 65.8 | 23 | 65.7 | 31 | 65.75 | 15 | 65.7 | 0 | 65.65 | -9 | 65.75 | -1 | 65.75 | -6 | 65.8 | 3 | 65.75 | 3 | 65.8 | -9 | 65.75 | -12 | 65.7 |
| 143.9778 | 2.3996 | 49 | 66.45 | 22 | 66.5 | 26 | 66.45 | 17 | 66.5 | -1 | 66.6 | -8 | 66.45 | 0 | 66.45 | -2 | 66.6 | 0 | 66.6 | 1 | 66.5 | -9 | 66.45 | -11 | 66.55 |
| 147.9778 | 2.4663 | 53 | 67.3 | 22 | 67.3 | 23 | 67.4 | 12 | 67.3 | 0 | 67.3 | -9 | 67.3 | -1 | 67.35 | -6 | 67.3 | -2 | 67.3 | 5 | 67.3 | -5 | 67.3 | -9 | 67.35 |
| 151.9778 | 2.5330 | 53 | 68.1 | 21 | 68.05 | 25 | 68 | 12 | 67.95 | 0 | 68.05 | -10 | 68 | 2 | 68 | -3 | 68.1 | 0 | 68 | 0 | 68.15 | -9 | 68.1 | -10 | 68 |
| 155.9778 | 2.5996 | 45 | 68.85 | 26 | 68.8 | 27 | 68.8 | 12 | 68.8 | 1 | 68.9 | -4 | 68.9 | 3 | 68.85 | -5 | 68.7 | 5 | 68.8 | 4 | 68.8 | -6 | 68.85 | -7 | 68.85 |
| 159.9778 | 2.6663 | 53 | 69.6 | 24 | 69.7 | 27 | 69.7 | 20 | 69.55 | 5 | 69.75 | 0 | 69.65 | 1 | 69.6 | -5 | 69.6 | 3 | 69.7 | 5 | 69.6 | -2 | 69.6 | -10 | 69.65 |
| 163.9778 | 2.7330 | 54 | 70.35 | 25 | 70.4 | 29 | 70.35 | 15 | 70.4 | 7 | 70.4 | 2 | 70.35 | 1 | 70.4 | -1 | 70.45 | 4 | 70.4 | 10 | 70.35 | -1 | 70.5 | -5 | 70.4 |
| 167.9778 | 2.7996 | 52 | 71.25 | 27 | 71.3 | 29 | 71.25 | 19 | 71.3 | 5 | 71.3 | 3 | 71.3 | 3 | 71.3 | -2 | 71.2 | 6 | 71.3 | 10 | 71.3 | 5 | 71.3 | -1 | 71.3 |
| 171.9778 | 2.8663 | 53 | 72.1 | 30 | 72 | 34 | 71.95 | 22 | 72 | 10 | 72 | 2 | 72.1 | 6 | 72 | 3 | 72 | 10 | 72 | 17 | 72.05 | 6 | 72.05 | 6 | 72 |
| 175.9778 | 2.9330 | 53 | 72.8 | 30 | 72.85 | 34 | 72.8 | 26 | 72.7 | 13 | 72.8 | 6 | 72.8 | 10 | 72.85 | 10 | 72.9 | 16 | 72.85 | 21 | 72.75 | 12 | 72.8 | 5 | 72.8 |
| 179.9778 | 2.9996 | 59 | 73.6 | 33 | 73.6 | 38 | 73.6 | 25 | 73.65 | 18 | 73.7 | 12 | 73.6 | 15 | 73.7 | 15 | 73.55 | 20 | 73.5 | 25 | 73.6 | 25 | 73.55 | 15 | 73.55 |
| 183.9778 | 3.0663 | 62 | 74.4 | 38 | 74.35 | 44 | 74.4 | 30 | 74.35 | 24 | 74.3 | 17 | 74.3 | 17 | 74.4 | 21 | 74.3 | 21 | 74.3 | 29 | 74.45 | 21 | 74.45 | 19 | 74.3 |
| 187.9778 | 3.1330 | 66 | 75.15 | 40 | 75.15 | 45 | 75.15 | 34 | 75.25 | 27 | 75.15 | 19 | 75.15 | 20 | 75.2 | 24 | 75.05 | 29 | 75.1 | 35 | 75.05 | 27 | 75.1 | 25 | 75.15 |
| 191.9778 | 3.1996 | 66 | 75.95 | 42 | 76 | 46 | 75.95 | 44 | 75.95 | 32 | 76 | 21 | 75.95 | 32 | 75.9 | 32 | 76 | 34 | 76 | 44 | 76 | 36 | 75.9 | 30 | 76.1 |
| 195.9778 | 3.2663 | 68 | 76.65 | 42 | 76.7 | 46 | 76.75 | 49 | 76.7 | 35 | 76.75 | 29 | 76.75 | 39 | 76.8 | 39 | 76.8 | 41 | 76.75 | 46 | 76.65 | 42 | 76.85 | 38 | 76.8 |
| 199.9778 | 3.3330 | 71 | 77.5 | 49 | 77.6 | 55 | 77.6 | 51 | 77.55 | 40 | 77.55 | 32 | 77.5 | 40 | 77.55 | 44 | 77.55 | 50 | 77.5 | 54 | 77.45 | 49 | 77.45 | 42 | 77.6 |
| 203.9778 | 3.3996 | 74 | 78.3 | 54 | 78.3 | 59 | 78.2 | 61 | 78.25 | 46 | 78.25 | 39 | 78.35 | 50 | 78.25 | 53 | 78.25 | 55 | 78.3 | 66 | 78.35 | 55 | 78.3 | 56 | 78.25 |
| 207.9778 | 3.4663 | 81 | 79.05 | 57 | 79.05 | 61 | 79.15 | 65 | 79.1 | 57 | 79.05 | 44 | 79.05 | 62 | 79.1 | 65 | 79.2 | 64 | 79.1 | 74 | 79.1 | 62 | 79 | 59 | 79.1 |
| 211.9778 | 3.5330 | 86 | 79.9 | 65 | 79.85 | 62 | 79.9 | 77 | 79.9 | 67 | 79.95 | 54 | 79.85 | 76 | 79.9 | 81 | 79.85 | 75 | 79.9 | 83 | 79.8 | 77 | 79.85 | 78 | 79.9 |
| 215.9778 | 3.5996 | 93 | 80.7 | 70 | 80.55 | 75 | 80.6 | 90 | 80.6 | 84 | 80.65 | 67 | 80.6 | 89 | 80.6 | 99 | 80.7 | 94 | 80.65 | 101 | 80.65 | 91 | 80.7 | 91 | 80.6 |
| 219.9778 | 3.6663 | 101 | 81.45 | 81 | 81.45 | 79 | 81.5 | 99 | 81.5 | 98 | 81.45 | 80 | 81.5 | 110 | 81.5 | 122 | 81.35 | 109 | 81.45 | 117 | 81.45 | 108 | 81.45 | 105 | 81.45 |
| 223.9778 | 3.7330 | 113 | 82.3 | 90 | 82.3 | 92 | 82.4 | 121 | 82.15 | 117 | 82.25 | 100 | 82.3 | 131 | 82.2 | 151 | 82.15 | 134 | 82.35 | 136 | 82.25 | 130 | 82.25 | 125 | 82.35 |
| 227.9778 | 3.7996 | 127 | 83 | 103 | 83.05 | 106 | 83 | 142 | 83.1 | 139 | 83.05 | 122 | 83 | 158 | 83 | 174 | 83.1 | 159 | 83.05 | 161 | 82.95 | 148 | 83.1 | 142 | 83.05 |
| 231.9778 | 3.8663 | 140 | 83.85 | 117 | 83.9 | 117 | 83.85 | 162 | 83.9 | 164 | 83.9 | 143 | 83.85 | 186 | 83.8 | 208 | 83.8 | 188 | 83.85 | 181 | 83.85 | 171 | 83.8 | 168 | 83.85 |
| 235.9778 | 3.9330 | 157 | 84.7 | 129 | 84.55 | 127 | 84.55 | 189 | 84.55 | 183 | 84.55 | 168 | 84.65 | 215 | 84.5 | 234 | 84.6 | 212 | 84.5 | 207 | 84.65 | 192 | 84.65 | 195 | 84.55 |
| 239.9778 | 3.9996 | 176 | 85.3 | 150 | 85.4 | 146 | 85.4 | 210 | 85.4 | 214 | 85.5 | 194 | 85.35 | 251 | 85.4 | 275 | 85.45 | 248 | 85.35 | 227 | 85.35 | 219 | 85.3 | 215 | 85.35 |
| 243.9778 | 4.0663 | 196 | 86.15 | 166 | 86.2 | 160 | 86.25 | 244 | 86.15 | 242 | 86.2 | 220 | 86.2 | 285 | 86.25 | 317 | 86.2 | 288 | 86.25 | 256 | 86.2 | 249 | 86.15 | 245 | 86.2 |
| 247.9778 | 4.1330 | 218 | 87.15 | 193 | 86.95 | 179 | 87 | 272 | 87 | 281 | 86.95 | 254 | 86.95 | 328 | 86.95 | 366 | 87.1 | 327 | 86.95 | 291 | 87.05 | 277 | 87.1 | 278 | 86.95 |
| 251.9778 | 4.1996 | 254 | 87.8 | 220 | 87.85 | 208 | 87.75 | 312 | 87.8 | 323 | 87.8 | 296 | 87.7 | 381 | 87.85 | 420 | 87.7 | 376 | 87.75 | 327 | 87.8 | 315 | 87.7 | 316 | 87.7 |
| 255.9778 | 4.2663 | 284 | 88.6 | 256 | 88.7 | 239 | 88.7 | 359 | 88.45 | 371 | 88.5 | 342 | 88.6 | 440 | 88.45 | 483 | 88.55 | 432 | 88.6 | 374 | 88.65 | 352 | 88.6 | 354 | 88.65 |
| 259.9778 | 4.3330 | 327 | 89.3 | 292 | 89.35 | 276 | 89.4 | 413 | 89.35 | 427 | 89.3 | 391 | 89.3 | 504 | 89.3 | 559 | 89.45 | 495 | 89.25 | 428 | 89.2 | 406 | 89.4 | 410 | 89.3 |
| 263.9778 | 4.3996 | 375 | 90.05 | 344 | 90.05 | 322 | 90.1 | 468 | 90.15 | 492 | 90.1 | 454 | 90.1 | 585 | 90.1 | 642 | 90.05 | 573 | 90.05 | 493 | 90.05 | 466 | 90.1 | 469 | 90.1 |

| **S3A: PASTING PROPERTIES RAW DATA PART 1 …continuation** | | | | | | | | | | | | | | | | | | | | | | | | | |
| --- | --- | --- | --- | --- | --- | --- | --- | --- | --- | --- | --- | --- | --- | --- | --- | --- | --- | --- | --- | --- | --- | --- | --- | --- | --- |
| **2019-15** | | **BROWN_UNF T1** | | **BROWN_UNF T2** | | **BROWN_UNF T3** | | **BROWN_FORT 1** | | **BROWN_FORT 2** | | **BROWN_FORT 3** | | **MILLED_UNF 1** | | **MILLED_UNF 2** | | **MILLED_UNF 3** | | **MILLED_FORT 1** | | **MILLED_FORT 2** | | **MILLED_FORT 3** | |
| time (sec) | time (min) | visc (cP) | temp (°C) | visc (cP) | temp (°C) | visc (cP) | temp (°C) | visc (cP) | temp (°C) | visc (cP) | temp (°C) | visc (cP) | temp (°C) | visc (cP) | temp (°C) | visc (cP) | temp (°C) | visc (cP) | temp (°C) | visc (cP) | temp (°C) | visc (cP) | temp (°C) | visc (cP) | temp (°C) |
| 267.9778 | 4.4663 | 432 | 90.85 | 400 | 90.75 | 371 | 90.8 | 538 | 90.85 | 564 | 90.75 | 522 | 90.9 | 669 | 90.85 | 740 | 90.9 | 662 | 90.8 | 563 | 90.8 | 536 | 90.9 | 537 | 90.8 |
| 271.9778 | 4.5330 | 498 | 91.6 | 466 | 91.65 | 430 | 91.6 | 615 | 91.65 | 648 | 91.65 | 599 | 91.6 | 776 | 91.7 | 850 | 91.65 | 762 | 91.65 | 639 | 91.55 | 616 | 91.5 | 613 | 91.6 |
| 275.9778 | 4.5996 | 568 | 92.4 | 541 | 92.5 | 503 | 92.5 | 701 | 92.5 | 744 | 92.45 | 685 | 92.55 | 884 | 92.55 | 979 | 92.4 | 880 | 92.5 | 739 | 92.45 | 704 | 92.45 | 705 | 92.55 |
| 279.9778 | 4.6663 | 650 | 93.25 | 620 | 93.15 | 576 | 93.25 | 807 | 93.15 | 852 | 93.2 | 788 | 93.15 | 1027 | 93.2 | 1135 | 93.25 | 1023 | 93.15 | 851 | 93.3 | 813 | 93.25 | 821 | 93.2 |
| 283.9778 | 4.7330 | 749 | 94 | 715 | 94 | 665 | 94.05 | 924 | 94.05 | 975 | 94 | 905 | 94.05 | 1196 | 94 | 1323 | 93.9 | 1182 | 94.05 | 1003 | 94.05 | 960 | 93.95 | 958 | 94.1 |
| 287.9778 | 4.7996 | 857 | 94.85 | 821 | 94.85 | 769 | 94.85 | 1054 | 94.8 | 1112 | 94.85 | 1027 | 94.85 | 1389 | 94.8 | 1539 | 94.75 | 1381 | 94.85 | 1171 | 94.75 | 1121 | 94.8 | 1126 | 94.9 |
| 291.9778 | 4.8663 | 971 | 95.35 | 943 | 95.3 | 876 | 95.3 | 1198 | 95.35 | 1264 | 95.3 | 1173 | 95.35 | 1603 | 95.3 | 1782 | 95.3 | 1599 | 95.4 | 1362 | 95.4 | 1314 | 95.45 | 1306 | 95.4 |
| 295.9778 | 4.9330 | 1097 | 95.3 | 1066 | 95.25 | 993 | 95.25 | 1352 | 95.25 | 1433 | 95.3 | 1329 | 95.3 | 1834 | 95.3 | 2049 | 95.25 | 1833 | 95.35 | 1557 | 95.35 | 1510 | 95.3 | 1510 | 95.3 |
| 299.9778 | 4.9996 | 1222 | 95 | 1199 | 95.1 | 1111 | 95.05 | 1520 | 95.1 | 1597 | 95.1 | 1484 | 94.95 | 2081 | 95.1 | 2317 | 95.1 | 2086 | 95.1 | 1773 | 94.7 | 1726 | 94.85 | 1711 | 94.95 |
| 303.9778 | 5.0663 | 1355 | 94.9 | 1328 | 94.95 | 1240 | 94.9 | 1688 | 94.9 | 1760 | 94.9 | 1643 | 94.7 | 2331 | 94.95 | 2559 | 94.95 | 2324 | 94.8 | 1985 | 94.6 | 1942 | 94.7 | 1925 | 94.75 |
| 307.9778 | 5.1330 | 1479 | 94.85 | 1459 | 94.9 | 1364 | 94.85 | 1850 | 94.8 | 1911 | 94.85 | 1806 | 94.65 | 2551 | 94.8 | 2784 | 94.9 | 2541 | 94.8 | 2176 | 94.7 | 2147 | 94.7 | 2124 | 94.75 |
| 311.9778 | 5.1996 | 1606 | 94.8 | 1583 | 94.85 | 1484 | 94.75 | 2007 | 94.7 | 2058 | 94.8 | 1962 | 94.7 | 2751 | 94.8 | 2985 | 94.85 | 2755 | 94.8 | 2360 | 94.75 | 2337 | 94.8 | 2313 | 94.75 |
| 315.9778 | 5.2663 | 1719 | 94.85 | 1694 | 94.85 | 1591 | 94.8 | 2153 | 94.75 | 2178 | 94.85 | 2104 | 94.85 | 2936 | 94.85 | 3164 | 94.9 | 2933 | 94.8 | 2534 | 94.9 | 2516 | 94.9 | 2492 | 94.8 |
| 319.9778 | 5.3330 | 1819 | 94.8 | 1790 | 94.9 | 1691 | 94.9 | 2271 | 94.85 | 2285 | 94.85 | 2234 | 94.9 | 3100 | 94.85 | 3307 | 94.85 | 3082 | 94.9 | 2686 | 95 | 2665 | 94.95 | 2663 | 94.95 |
| 323.9778 | 5.3996 | 1902 | 94.9 | 1872 | 94.9 | 1784 | 94.95 | 2382 | 94.9 | 2378 | 94.95 | 2343 | 95 | 3242 | 94.9 | 3430 | 94.9 | 3216 | 94.9 | 2828 | 95 | 2815 | 95 | 2812 | 94.95 |
| 327.9778 | 5.4663 | 1977 | 94.9 | 1948 | 95.05 | 1857 | 94.95 | 2471 | 95 | 2455 | 95 | 2436 | 95.05 | 3358 | 94.95 | 3553 | 94.9 | 3340 | 94.95 | 2959 | 95.15 | 2949 | 95.05 | 2943 | 94.95 |
| 331.9778 | 5.5330 | 2043 | 95 | 2011 | 94.9 | 1923 | 94.95 | 2545 | 94.95 | 2517 | 94.95 | 2515 | 95.05 | 3452 | 95 | 3670 | 95 | 3455 | 94.95 | 3094 | 95.05 | 3084 | 95 | 3060 | 95.05 |
| 335.9778 | 5.5996 | 2102 | 95 | 2062 | 95 | 1974 | 95.05 | 2608 | 95 | 2564 | 94.95 | 2577 | 95.1 | 3536 | 94.95 | 3764 | 94.95 | 3544 | 94.95 | 3227 | 95.1 | 3219 | 95 | 3174 | 95.1 |
| 339.9778 | 5.6663 | 2150 | 95 | 2106 | 95 | 2021 | 95 | 2650 | 95.05 | 2596 | 95.1 | 2634 | 95.1 | 3616 | 95 | 3821 | 94.9 | 3611 | 94.95 | 3352 | 95.05 | 3337 | 95.1 | 3291 | 95.1 |
| 343.9778 | 5.7330 | 2193 | 95.05 | 2138 | 94.95 | 2065 | 95 | 2692 | 95 | 2618 | 95 | 2676 | 95.1 | 3687 | 95.05 | 3847 | 95 | 3655 | 95 | 3445 | 95.05 | 3429 | 95.05 | 3406 | 95.05 |
| 347.9778 | 5.7996 | 2226 | 95 | 2167 | 95.05 | 2094 | 95.05 | 2718 | 95.1 | 2640 | 95 | 2712 | 95.05 | 3735 | 95.05 | 3874 | 95.1 | 3694 | 95 | 3520 | 95.05 | 3503 | 95.05 | 3509 | 95.05 |
| 351.9778 | 5.8663 | 2254 | 95.05 | 2191 | 95.05 | 2122 | 95.05 | 2741 | 95.05 | 2649 | 95.05 | 2738 | 95.05 | 3770 | 95 | 3894 | 95.05 | 3719 | 95.05 | 3582 | 95.05 | 3563 | 95.05 | 3588 | 95 |
| 355.9778 | 5.9330 | 2274 | 95.05 | 2206 | 95.05 | 2147 | 95.05 | 2749 | 95.05 | 2652 | 95.1 | 2758 | 95.05 | 3794 | 95.05 | 3892 | 95.05 | 3736 | 95 | 3633 | 95.05 | 3611 | 95.05 | 3644 | 95.05 |
| 359.9778 | 5.9996 | 2291 | 95.05 | 2209 | 95.05 | 2158 | 95.05 | 2759 | 95 | 2653 | 95.05 | 2766 | 95.05 | 3803 | 95.05 | 3884 | 95.05 | 3734 | 95.05 | 3669 | 95.05 | 3651 | 95.05 | 3691 | 95.1 |
| 363.9778 | 6.0663 | 2294 | 95 | 2215 | 95.05 | 2171 | 95.05 | 2755 | 95.05 | 2641 | 95 | 2768 | 95 | 3804 | 95.05 | 3863 | 95.05 | 3729 | 95.05 | 3681 | 95 | 3671 | 95 | 3726 | 95.05 |
| 367.9778 | 6.1330 | 2301 | 95.05 | 2214 | 95.05 | 2183 | 95.05 | 2751 | 95.05 | 2631 | 95.05 | 2766 | 95.05 | 3798 | 95.05 | 3831 | 95.05 | 3706 | 95.05 | 3697 | 95.05 | 3672 | 95 | 3748 | 95 |
| 371.9778 | 6.1996 | 2301 | 95.05 | 2217 | 95.05 | 2184 | 95.05 | 2741 | 95 | 2618 | 95 | 2760 | 95.05 | 3782 | 95 | 3801 | 95.05 | 3686 | 95 | 3692 | 95.05 | 3668 | 95.05 | 3760 | 95 |
| 375.9778 | 6.2663 | 2299 | 95.05 | 2206 | 95 | 2181 | 95.1 | 2730 | 95 | 2600 | 95.05 | 2747 | 95.05 | 3760 | 95.05 | 3758 | 95 | 3658 | 95 | 3678 | 95.1 | 3650 | 95 | 3760 | 94.9 |
| 379.9778 | 6.3330 | 2296 | 94.95 | 2212 | 95 | 2187 | 95 | 2712 | 95.05 | 2580 | 95 | 2739 | 95.05 | 3736 | 95.1 | 3715 | 95.05 | 3622 | 95 | 3656 | 95.05 | 3633 | 95 | 3755 | 94.95 |
| 383.9778 | 6.3996 | 2292 | 95 | 2199 | 95.05 | 2170 | 94.95 | 2696 | 95 | 2563 | 95.05 | 2718 | 95 | 3696 | 95 | 3664 | 95.05 | 3583 | 95 | 3630 | 95.05 | 3604 | 95.1 | 3738 | 95 |
| 387.9778 | 6.4663 | 2283 | 95 | 2193 | 95 | 2167 | 95 | 2675 | 95 | 2542 | 95.05 | 2699 | 95.05 | 3658 | 95 | 3622 | 95 | 3547 | 95 | 3595 | 94.95 | 3577 | 95.05 | 3714 | 95 |
| 391.9778 | 6.5330 | 2275 | 95 | 2183 | 95 | 2162 | 95 | 2653 | 95.05 | 2518 | 95 | 2675 | 95.1 | 3617 | 95.05 | 3580 | 95 | 3500 | 94.95 | 3557 | 95 | 3541 | 95 | 3682 | 95 |
| 395.9778 | 6.5996 | 2263 | 95 | 2169 | 95 | 2152 | 95 | 2632 | 95.05 | 2496 | 95.05 | 2655 | 95 | 3575 | 95.05 | 3531 | 95 | 3467 | 95 | 3521 | 95 | 3502 | 95.1 | 3656 | 95 |
| 399.9778 | 6.6663 | 2251 | 95 | 2155 | 95 | 2142 | 95 | 2607 | 95 | 2476 | 95.05 | 2628 | 94.95 | 3533 | 95.05 | 3480 | 95.05 | 3421 | 95.05 | 3480 | 95.05 | 3459 | 95.05 | 3616 | 95 |
| 403.9778 | 6.7330 | 2238 | 94.95 | 2149 | 95.05 | 2138 | 95.05 | 2584 | 95.05 | 2454 | 95 | 2609 | 95.05 | 3492 | 95 | 3433 | 95.1 | 3378 | 95 | 3438 | 95 | 3420 | 95 | 3575 | 95 |
| 407.9778 | 6.7996 | 2228 | 95 | 2138 | 95.05 | 2129 | 95.1 | 2560 | 95.05 | 2435 | 95.05 | 2587 | 95 | 3445 | 95.05 | 3381 | 95.05 | 3333 | 95 | 3397 | 95 | 3380 | 95.05 | 3534 | 95 |
| 411.9778 | 6.8663 | 2208 | 95 | 2126 | 95.05 | 2117 | 95.05 | 2539 | 95.05 | 2413 | 95.05 | 2562 | 95 | 3403 | 94.95 | 3340 | 95.05 | 3304 | 95.05 | 3353 | 94.95 | 3338 | 95.05 | 3492 | 95 |
| 415.9778 | 6.9330 | 2196 | 95.05 | 2116 | 95.1 | 2106 | 94.95 | 2521 | 95 | 2392 | 95 | 2540 | 95 | 3353 | 95.05 | 3294 | 95.05 | 3251 | 94.95 | 3316 | 95 | 3297 | 94.95 | 3451 | 95.05 |
| 419.9778 | 6.9996 | 2185 | 95.05 | 2101 | 94.95 | 2095 | 95.05 | 2498 | 95 | 2373 | 95 | 2517 | 95 | 3311 | 94.95 | 3257 | 94.95 | 3216 | 95.05 | 3275 | 95.05 | 3256 | 95 | 3402 | 95.1 |
| 423.9778 | 7.0663 | 2169 | 95.05 | 2092 | 95.05 | 2084 | 94.95 | 2475 | 94.95 | 2355 | 95.05 | 2491 | 94.95 | 3272 | 95.05 | 3214 | 95.05 | 3175 | 95 | 3233 | 95 | 3214 | 95.05 | 3362 | 95 |
| 427.9778 | 7.1330 | 2157 | 95 | 2076 | 95 | 2080 | 95.05 | 2452 | 94.95 | 2333 | 94.95 | 2469 | 94.95 | 3231 | 95 | 3182 | 94.95 | 3133 | 95 | 3194 | 95 | 3179 | 95 | 3321 | 95.1 |
| 431.9778 | 7.1996 | 2145 | 95.05 | 2072 | 94.95 | 2072 | 95.05 | 2430 | 95.05 | 2313 | 95.05 | 2450 | 95 | 3197 | 95 | 3139 | 94.95 | 3109 | 95 | 3153 | 95.05 | 3138 | 95 | 3282 | 95 |
| 435.9778 | 7.2663 | 2135 | 95 | 2057 | 95 | 2056 | 95.05 | 2410 | 95 | 2302 | 95.1 | 2426 | 94.95 | 3158 | 95.05 | 3105 | 95 | 3057 | 95.05 | 3114 | 95.05 | 3108 | 95 | 3248 | 95 |
| 439.9778 | 7.3330 | 2121 | 95.05 | 2050 | 95 | 2047 | 95 | 2392 | 95 | 2281 | 95.05 | 2406 | 95 | 3122 | 95 | 3069 | 95.05 | 3030 | 95.05 | 3082 | 95 | 3074 | 95 | 3203 | 95.05 |
| 443.9778 | 7.3996 | 2109 | 94.9 | 2037 | 94.85 | 2035 | 94.7 | 2372 | 94.65 | 2266 | 94.9 | 2388 | 94.7 | 3089 | 94.9 | 3036 | 94.75 | 2996 | 94.7 | 3054 | 94.75 | 3044 | 94.75 | 3171 | 94.9 |
| 447.9778 | 7.4663 | 2098 | 93.55 | 2030 | 93.6 | 2023 | 93.5 | 2359 | 93.65 | 2247 | 93.55 | 2370 | 93.65 | 3055 | 93.9 | 3009 | 93.65 | 2969 | 93.55 | 3020 | 93.6 | 3009 | 93.55 | 3129 | 93.55 |
| 451.9778 | 7.5330 | 2089 | 92.65 | 2020 | 92.65 | 2020 | 92.65 | 2339 | 92.7 | 2235 | 92.6 | 2358 | 92.7 | 3026 | 92.55 | 2987 | 92.6 | 2948 | 92.75 | 2994 | 92.65 | 2985 | 92.6 | 3100 | 92.65 |
| 455.9778 | 7.5996 | 2079 | 91.8 | 2014 | 91.8 | 2016 | 91.85 | 2328 | 91.8 | 2223 | 91.75 | 2341 | 91.7 | 3000 | 91.8 | 2965 | 91.85 | 2919 | 91.9 | 2975 | 91.8 | 2967 | 91.9 | 3075 | 91.7 |
| 459.9778 | 7.6663 | 2078 | 91 | 2012 | 91.05 | 2013 | 91.15 | 2318 | 91.05 | 2210 | 91.1 | 2328 | 90.9 | 2982 | 91 | 2949 | 91.05 | 2913 | 91.15 | 2956 | 91 | 2943 | 91 | 3058 | 91 |
| 463.9778 | 7.7330 | 2073 | 90.25 | 2005 | 90.2 | 2008 | 90.1 | 2306 | 90.1 | 2208 | 90.15 | 2318 | 90.2 | 2962 | 90.1 | 2935 | 90.15 | 2892 | 90.1 | 2935 | 90.15 | 2924 | 90.25 | 3042 | 90.2 |
| 467.9778 | 7.7996 | 2072 | 89.3 | 2001 | 89.35 | 2003 | 89.35 | 2305 | 89.3 | 2201 | 89.25 | 2310 | 89.45 | 2950 | 89.25 | 2923 | 89.4 | 2883 | 89.35 | 2922 | 89.4 | 2911 | 89.35 | 3020 | 89.35 |
| 471.9778 | 7.8663 | 2060 | 88.55 | 1999 | 88.55 | 2003 | 88.65 | 2293 | 88.55 | 2193 | 88.55 | 2301 | 88.75 | 2933 | 88.6 | 2914 | 88.55 | 2868 | 88.6 | 2911 | 88.5 | 2899 | 88.5 | 3008 | 88.7 |
| 475.9778 | 7.9330 | 2058 | 87.75 | 1998 | 87.75 | 2019 | 87.7 | 2291 | 87.7 | 2190 | 87.8 | 2293 | 87.8 | 2921 | 87.7 | 2902 | 87.75 | 2860 | 87.75 | 2904 | 87.85 | 2891 | 87.75 | 2994 | 87.75 |
| 479.9778 | 7.9996 | 2057 | 86.8 | 1997 | 86.9 | 1999 | 86.8 | 2289 | 87.05 | 2188 | 86.8 | 2291 | 86.9 | 2914 | 87 | 2898 | 87.05 | 2852 | 86.9 | 2894 | 86.95 | 2884 | 87.05 | 2987 | 86.8 |
| 483.9778 | 8.0663 | 2053 | 86.1 | 1999 | 86.05 | 1995 | 86.2 | 2274 | 86.1 | 2186 | 86.15 | 2289 | 86.15 | 2901 | 86.15 | 2894 | 86.05 | 2843 | 86.1 | 2889 | 86.2 | 2874 | 86 | 2975 | 86.1 |
| 487.9778 | 8.1330 | 2048 | 85.2 | 1991 | 85.25 | 1989 | 85.2 | 2272 | 85.25 | 2182 | 85.25 | 2284 | 85.25 | 2899 | 85.35 | 2887 | 85.3 | 2844 | 85.3 | 2880 | 85.45 | 2869 | 85.2 | 2972 | 85.35 |
| 491.9778 | 8.1996 | 2048 | 84.45 | 1988 | 84.55 | 1994 | 84.5 | 2266 | 84.6 | 2179 | 84.6 | 2280 | 84.55 | 2892 | 84.6 | 2888 | 84.45 | 2840 | 84.6 | 2877 | 84.45 | 2868 | 84.5 | 2966 | 84.65 |
| 495.9778 | 8.2663 | 2045 | 83.75 | 1984 | 83.75 | 1988 | 83.7 | 2259 | 83.8 | 2177 | 83.7 | 2274 | 83.75 | 2894 | 83.8 | 2880 | 83.65 | 2833 | 83.65 | 2878 | 83.75 | 2865 | 83.85 | 2965 | 83.7 |
| 499.9778 | 8.3330 | 2039 | 83 | 1986 | 82.8 | 1994 | 83 | 2256 | 83 | 2173 | 82.85 | 2271 | 82.95 | 2886 | 82.9 | 2881 | 83 | 2840 | 82.9 | 2880 | 83 | 2865 | 83 | 2965 | 82.8 |
| 503.9778 | 8.3996 | 2040 | 82.15 | 1981 | 82.1 | 1989 | 82.25 | 2255 | 82.2 | 2171 | 82.2 | 2271 | 82.15 | 2889 | 82.2 | 2884 | 82.05 | 2836 | 82.2 | 2874 | 82.1 | 2865 | 82.1 | 2966 | 82.15 |
| 507.9778 | 8.4663 | 2030 | 81.5 | 1988 | 81.35 | 1989 | 81.45 | 2250 | 81.3 | 2167 | 81.25 | 2267 | 81.3 | 2890 | 81.35 | 2887 | 81.35 | 2834 | 81.2 | 2879 | 81.35 | 2867 | 81.5 | 2959 | 81.3 |
| 511.9778 | 8.5330 | 2033 | 80.7 | 1983 | 80.4 | 1983 | 80.5 | 2248 | 80.7 | 2167 | 80.45 | 2267 | 80.55 | 2895 | 80.5 | 2887 | 80.55 | 2840 | 80.5 | 2881 | 80.45 | 2867 | 80.7 | 2960 | 80.45 |
| 515.9778 | 8.5996 | 2032 | 79.85 | 1984 | 79.75 | 1987 | 79.85 | 2248 | 79.75 | 2170 | 79.75 | 2267 | 79.75 | 2895 | 79.7 | 2896 | 79.75 | 2844 | 79.7 | 2889 | 79.75 | 2874 | 79.8 | 2957 | 79.75 |
| 519.9778 | 8.6663 | 2031 | 79 | 1984 | 78.9 | 1984 | 78.9 | 2252 | 78.9 | 2169 | 79 | 2264 | 79 | 2904 | 78.95 | 2904 | 79.15 | 2850 | 79.05 | 2896 | 79.15 | 2882 | 79.05 | 2967 | 78.95 |
| 523.9778 | 8.7330 | 2032 | 78.15 | 1987 | 78.25 | 1986 | 78.25 | 2254 | 78.15 | 2174 | 78.35 | 2266 | 78.15 | 2909 | 78.25 | 2914 | 78.2 | 2857 | 78.35 | 2904 | 78.1 | 2886 | 78.15 | 2975 | 78.15 |
| 527.9778 | 8.7996 | 2043 | 77.45 | 1990 | 77.55 | 1988 | 77.45 | 2260 | 77.4 | 2182 | 77.5 | 2275 | 77.4 | 2920 | 77.45 | 2928 | 77.4 | 2877 | 77.5 | 2916 | 77.45 | 2899 | 77.5 | 2987 | 77.35 |
| 531.9778 | 8.8663 | 2045 | 76.75 | 1998 | 76.75 | 1994 | 76.65 | 2263 | 76.55 | 2193 | 76.65 | 2284 | 76.65 | 2938 | 76.5 | 2949 | 76.8 | 2892 | 76.7 | 2927 | 76.65 | 2915 | 76.75 | 2994 | 76.6 |
| 535.9778 | 8.9330 | 2050 | 75.7 | 2002 | 75.95 | 2008 | 75.9 | 2270 | 75.9 | 2208 | 76 | 2294 | 75.95 | 2959 | 75.9 | 2974 | 75.85 | 2916 | 75.9 | 2942 | 75.8 | 2931 | 75.8 | 3010 | 76.1 |
| 539.9778 | 8.9996 | 2062 | 75.05 | 2014 | 75.1 | 2015 | 75.1 | 2286 | 75.05 | 2219 | 75.05 | 2305 | 75.15 | 2987 | 75.05 | 3005 | 75.2 | 2936 | 75.15 | 2962 | 75.15 | 2958 | 75.1 | 3033 | 75.05 |
| 543.9778 | 9.0663 | 2079 | 74.2 | 2033 | 74.2 | 2022 | 74.2 | 2306 | 74.4 | 2236 | 74.3 | 2321 | 74.25 | 3008 | 74.25 | 3040 | 74.3 | 2969 | 74.25 | 2990 | 74.25 | 2979 | 74.25 | 3053 | 74.35 |
| **S3A: PASTING PROPERTIES RAW DATA PART 1…continuation** | | | | | | | | | | | | | | | | | | | | | | | | | |
| **2019-15** | | **BROWN_UNF T1** | | **BROWN_UNF T2** | | **BROWN_UNF T3** | | **BROWN_FORT 1** | | **BROWN_FORT 2** | | **BROWN_FORT 3** | | **MILLED_UNF 1** | | **MILLED_UNF 2** | | **MILLED_UNF 3** | | **MILLED_FORT 1** | | **MILLED_FORT 2** | | **MILLED_FORT 3** | |
| time (sec) | time (min) | visc (cP) | temp (°C) | visc (cP) | temp (°C) | visc (cP) | temp (°C) | visc (cP) | temp (°C) | visc (cP) | temp (°C) | visc (cP) | temp (°C) | visc (cP) | temp (°C) | visc (cP) | temp (°C) | visc (cP) | temp (°C) | visc (cP) | temp (°C) | visc (cP) | temp (°C) | visc (cP) | temp (°C) |
| 547.9778 | 9.1330 | 2086 | 73.5 | 2047 | 73.55 | 2041 | 73.55 | 2322 | 73.45 | 2260 | 73.55 | 2340 | 73.6 | 3033 | 73.55 | 3074 | 73.55 | 2997 | 73.55 | 3020 | 73.65 | 3011 | 73.4 | 3080 | 73.6 |
| 551.9778 | 9.1996 | 2101 | 72.75 | 2058 | 72.7 | 2054 | 72.7 | 2340 | 72.75 | 2283 | 72.75 | 2360 | 72.8 | 3070 | 72.7 | 3104 | 72.8 | 3028 | 72.7 | 3045 | 72.75 | 3036 | 72.8 | 3104 | 72.75 |
| 555.9778 | 9.2663 | 2121 | 71.85 | 2072 | 72 | 2074 | 72 | 2364 | 72 | 2306 | 72.05 | 2379 | 71.85 | 3097 | 72 | 3146 | 71.95 | 3051 | 71.95 | 3080 | 71.9 | 3069 | 71.9 | 3133 | 72.1 |
| 559.9778 | 9.3330 | 2136 | 71.25 | 2094 | 71.25 | 2105 | 71.15 | 2386 | 71.25 | 2333 | 71.1 | 2402 | 71.2 | 3140 | 71.25 | 3186 | 71.2 | 3099 | 71.2 | 3112 | 71.3 | 3099 | 71.25 | 3161 | 71.15 |
| 563.9778 | 9.3996 | 2153 | 70.45 | 2116 | 70.3 | 2115 | 70.25 | 2415 | 70.35 | 2357 | 70.35 | 2429 | 70.35 | 3173 | 70.35 | 3225 | 70.35 | 3134 | 70.2 | 3147 | 70.3 | 3131 | 70.45 | 3187 | 70.35 |
| 567.9778 | 9.4663 | 2169 | 69.55 | 2135 | 69.7 | 2123 | 69.65 | 2441 | 69.6 | 2386 | 69.65 | 2459 | 69.6 | 3209 | 69.7 | 3272 | 69.55 | 3169 | 69.6 | 3184 | 69.55 | 3171 | 69.55 | 3230 | 69.65 |
| 571.9778 | 9.5330 | 2199 | 68.9 | 2162 | 68.75 | 2149 | 68.75 | 2471 | 68.8 | 2421 | 68.75 | 2489 | 68.9 | 3257 | 68.8 | 3323 | 68.95 | 3216 | 68.8 | 3222 | 68.85 | 3206 | 68.95 | 3266 | 68.75 |
| 575.9778 | 9.5996 | 2222 | 68.05 | 2189 | 68 | 2177 | 68.05 | 2502 | 68.05 | 2450 | 68 | 2520 | 67.95 | 3303 | 68.1 | 3383 | 68.1 | 3275 | 67.95 | 3267 | 68.05 | 3255 | 68 | 3302 | 68 |
| 579.9778 | 9.6663 | 2252 | 67.3 | 2225 | 67.3 | 2208 | 67.35 | 2538 | 67.3 | 2490 | 67.35 | 2555 | 67.3 | 3372 | 67.3 | 3445 | 67.3 | 3341 | 67.35 | 3316 | 67.3 | 3297 | 67.25 | 3350 | 67.3 |
| 583.9778 | 9.7330 | 2284 | 66.55 | 2260 | 66.5 | 2239 | 66.5 | 2576 | 66.5 | 2530 | 66.6 | 2591 | 66.55 | 3425 | 66.5 | 3516 | 66.6 | 3401 | 66.5 | 3371 | 66.4 | 3360 | 66.65 | 3401 | 66.5 |
| 587.9778 | 9.7996 | 2323 | 65.65 | 2294 | 65.85 | 2297 | 65.85 | 2618 | 65.7 | 2573 | 65.9 | 2638 | 65.7 | 3488 | 65.8 | 3587 | 65.8 | 3470 | 65.8 | 3431 | 65.7 | 3418 | 65.75 | 3456 | 65.9 |
| 591.9778 | 9.8663 | 2358 | 64.85 | 2333 | 64.95 | 2313 | 65 | 2669 | 64.95 | 2625 | 64.9 | 2677 | 65 | 3559 | 64.9 | 3665 | 64.9 | 3538 | 64.95 | 3501 | 64.95 | 3485 | 65.05 | 3516 | 64.95 |
| 595.9778 | 9.9330 | 2406 | 64.15 | 2378 | 64.15 | 2353 | 64.05 | 2713 | 64 | 2672 | 64.15 | 2728 | 64.15 | 3638 | 64.05 | 3745 | 64.15 | 3617 | 64.1 | 3563 | 64.15 | 3553 | 64.15 | 3583 | 64.15 |
| 599.9778 | 9.9996 | 2447 | 63.25 | 2419 | 63.5 | 2398 | 63.4 | 2760 | 63.35 | 2718 | 63.4 | 2775 | 63.45 | 3704 | 63.4 | 3826 | 63.35 | 3686 | 63.45 | 3631 | 63.35 | 3623 | 63.3 | 3649 | 63.45 |
| 603.9778 | 10.0663 | 2486 | 62.7 | 2465 | 62.55 | 2435 | 62.5 | 2812 | 62.65 | 2772 | 62.6 | 2824 | 62.6 | 3784 | 62.55 | 3907 | 62.65 | 3765 | 62.6 | 3707 | 62.65 | 3697 | 62.7 | 3719 | 62.65 |
| 607.9778 | 10.1330 | 2527 | 61.8 | 2503 | 61.8 | 2480 | 61.85 | 2861 | 61.95 | 2823 | 61.75 | 2874 | 61.75 | 3853 | 61.9 | 3988 | 61.8 | 3838 | 61.7 | 3778 | 61.9 | 3769 | 61.75 | 3793 | 61.75 |
| 611.9778 | 10.1996 | 2568 | 61.1 | 2553 | 61.05 | 2519 | 61.1 | 2911 | 61.1 | 2882 | 61.05 | 2925 | 61.1 | 3928 | 61.1 | 4065 | 60.95 | 3917 | 61.1 | 3852 | 61.15 | 3840 | 61 | 3858 | 61.05 |
| 615.9778 | 10.2663 | 2613 | 60.3 | 2595 | 60.25 | 2567 | 60.25 | 2962 | 60.3 | 2934 | 60.3 | 2978 | 60.2 | 4003 | 60.25 | 4145 | 60.3 | 3994 | 60.2 | 3927 | 60.2 | 3907 | 60.4 | 3925 | 60.3 |
| 619.9778 | 10.3330 | 2659 | 59.3 | 2648 | 59.5 | 2611 | 59.55 | 3010 | 59.45 | 2985 | 59.6 | 3028 | 59.35 | 4080 | 59.6 | 4223 | 59.5 | 4068 | 59.55 | 3997 | 59.4 | 3981 | 59.4 | 3996 | 59.5 |
| 623.9778 | 10.3996 | 2707 | 58.7 | 2694 | 58.7 | 2649 | 58.7 | 3063 | 58.7 | 3036 | 58.7 | 3079 | 58.75 | 4153 | 58.65 | 4302 | 58.55 | 4150 | 58.75 | 4069 | 58.7 | 4048 | 58.75 | 4060 | 58.65 |
| 627.9778 | 10.4663 | 2748 | 57.85 | 2737 | 57.95 | 2693 | 57.9 | 3117 | 57.75 | 3091 | 57.9 | 3133 | 57.85 | 4222 | 57.85 | 4378 | 57.9 | 4214 | 57.8 | 4136 | 57.9 | 4117 | 57.9 | 4131 | 57.95 |
| 631.9778 | 10.5330 | 2792 | 57.1 | 2784 | 57.2 | 2743 | 57.2 | 3165 | 57.15 | 3146 | 57.15 | 3188 | 57.2 | 4293 | 57.1 | 4453 | 57.15 | 4290 | 57.2 | 4201 | 57 | 4181 | 57.1 | 4195 | 57.2 |
| 635.9778 | 10.5996 | 2838 | 56.45 | 2825 | 56.4 | 2792 | 56.3 | 3218 | 56.3 | 3194 | 56.45 | 3238 | 56.45 | 4361 | 56.3 | 4526 | 56.45 | 4361 | 56.35 | 4267 | 56.4 | 4252 | 56.45 | 4258 | 56.45 |
| 639.9778 | 10.6663 | 2881 | 55.5 | 2877 | 55.55 | 2830 | 55.55 | 3267 | 55.65 | 3243 | 55.5 | 3294 | 55.5 | 4434 | 55.7 | 4597 | 55.65 | 4433 | 55.5 | 4330 | 55.6 | 4312 | 55.55 | 4319 | 55.5 |
| 643.9778 | 10.7330 | 2927 | 54.75 | 2919 | 54.8 | 2875 | 54.85 | 3319 | 54.85 | 3291 | 54.8 | 3347 | 54.9 | 4504 | 54.85 | 4673 | 54.8 | 4497 | 54.8 | 4387 | 55 | 4370 | 54.75 | 4382 | 54.85 |
| 647.9778 | 10.7996 | 2975 | 54 | 2962 | 53.95 | 2913 | 54 | 3372 | 53.9 | 3339 | 53.95 | 3395 | 53.95 | 4568 | 54 | 4739 | 53.95 | 4567 | 53.95 | 4448 | 54.05 | 4438 | 54 | 4439 | 54 |
| 651.9778 | 10.8663 | 3012 | 53.2 | 3010 | 53.3 | 2950 | 53.3 | 3419 | 53.25 | 3394 | 53.3 | 3445 | 53.2 | 4637 | 53.25 | 4808 | 53.25 | 4638 | 53.25 | 4509 | 53.2 | 4495 | 53.2 | 4503 | 53.25 |
| 655.9778 | 10.9330 | 3058 | 52.6 | 3052 | 52.4 | 2986 | 52.4 | 3472 | 52.5 | 3451 | 52.45 | 3495 | 52.5 | 4699 | 52.5 | 4881 | 52.35 | 4705 | 52.45 | 4568 | 52.5 | 4552 | 52.6 | 4556 | 52.5 |
| 659.9778 | 10.9996 | 3108 | 51.7 | 3096 | 51.7 | 3030 | 51.7 | 3528 | 51.6 | 3506 | 51.65 | 3548 | 51.65 | 4767 | 51.65 | 4942 | 51.75 | 4763 | 51.7 | 4626 | 51.75 | 4601 | 51.65 | 4611 | 51.6 |
| 663.9778 | 11.0663 | 3144 | 50.85 | 3138 | 50.95 | 3080 | 50.9 | 3582 | 50.9 | 3556 | 50.9 | 3600 | 50.95 | 4826 | 50.9 | 5011 | 50.85 | 4820 | 51 | 4678 | 50.85 | 4650 | 50.85 | 4662 | 50.9 |
| 667.9778 | 11.1330 | 3192 | 50.2 | 3180 | 50.15 | 3121 | 50.1 | 3632 | 50.15 | 3614 | 50.15 | 3648 | 50.2 | 4884 | 50.1 | 5077 | 50.25 | 4887 | 50.2 | 4729 | 50.1 | 4696 | 50.2 | 4717 | 50.15 |
| 671.9778 | 11.1996 | 3231 | 50.1 | 3229 | 50 | 3161 | 49.95 | 3689 | 50.1 | 3660 | 50.05 | 3696 | 50.1 | 4943 | 49.95 | 5138 | 50 | 4944 | 50.05 | 4783 | 49.95 | 4746 | 50.15 | 4763 | 50.15 |
| 675.9778 | 11.2663 | 3267 | 50.1 | 3288 | 50.1 | 3208 | 50.1 | 3741 | 50.1 | 3708 | 50.2 | 3746 | 50.1 | 4997 | 50.15 | 5195 | 50.15 | 5003 | 50.05 | 4828 | 50.1 | 4787 | 50.15 | 4815 | 50.15 |
| 679.9778 | 11.3330 | 3307 | 50.1 | 3323 | 50.2 | 3253 | 50.25 | 3786 | 50.2 | 3752 | 50.2 | 3793 | 50.2 | 5045 | 50.25 | 5249 | 50.2 | 5055 | 50.15 | 4867 | 50.2 | 4830 | 50.15 | 4853 | 50.2 |
| 683.9778 | 11.3996 | 3346 | 50.15 | 3363 | 50.15 | 3280 | 50.2 | 3831 | 50 | 3797 | 50 | 3845 | 50.15 | 5091 | 50.2 | 5297 | 50.1 | 5110 | 50.15 | 4907 | 50.15 | 4865 | 50.05 | 4897 | 50.1 |
| 687.9778 | 11.4663 | 3384 | 50.05 | 3396 | 50.05 | 3312 | 50.05 | 3869 | 50.05 | 3837 | 50.05 | 3888 | 50.1 | 5129 | 50 | 5341 | 50 | 5154 | 50.15 | 4938 | 50.05 | 4897 | 50.05 | 4937 | 50.1 |
| 691.9778 | 11.5330 | 3412 | 50 | 3434 | 50 | 3346 | 50 | 3909 | 50.05 | 3877 | 50.1 | 3923 | 50 | 5167 | 49.95 | 5379 | 50 | 5194 | 50.15 | 4977 | 50.05 | 4931 | 50.05 | 4979 | 50.05 |
| 695.9778 | 11.5996 | 3450 | 50 | 3467 | 50 | 3382 | 50.05 | 3944 | 50 | 3919 | 49.95 | 3968 | 49.9 | 5210 | 50 | 5420 | 50.1 | 5233 | 50 | 5001 | 50.05 | 4969 | 50 | 5009 | 50.05 |
| 699.9778 | 11.6663 | 3482 | 50 | 3500 | 50.1 | 3419 | 50.1 | 3977 | 50.05 | 3948 | 50 | 4007 | 50 | 5248 | 50.1 | 5458 | 50.05 | 5263 | 50 | 5035 | 50.1 | 5000 | 50 | 5045 | 50.05 |
| 703.9778 | 11.7330 | 3513 | 50.1 | 3520 | 50.05 | 3439 | 50 | 4012 | 50.05 | 3985 | 50 | 4037 | 50.1 | 5283 | 50.05 | 5489 | 50 | 5296 | 50 | 5068 | 50.1 | 5024 | 50 | 5070 | 50.1 |
| 707.9778 | 11.7996 | 3538 | 50.1 | 3557 | 49.95 | 3459 | 49.95 | 4041 | 50.05 | 4035 | 50.05 | 4073 | 50.05 | 5321 | 50.05 | 5519 | 49.9 | 5327 | 50.05 | 5094 | 50.05 | 5054 | 50 | 5101 | 50.05 |
| 711.9778 | 11.8663 | 3565 | 50 | 3571 | 50 | 3486 | 50 | 4079 | 50 | 4081 | 50.15 | 4097 | 50.05 | 5366 | 49.95 | 5551 | 49.95 | 5358 | 50.05 | 5119 | 49.95 | 5078 | 49.95 | 5141 | 49.95 |
| 715.9778 | 11.9330 | 3589 | 49.9 | 3604 | 50.05 | 3511 | 50 | 4132 | 50.05 | 4102 | 50.05 | 4128 | 49.95 | 5401 | 49.95 | 5579 | 50.05 | 5380 | 50 | 5147 | 50 | 5102 | 50 | 5165 | 49.95 |
| 719.9778 | 11.9996 | 3610 | 49.9 | 3628 | 50.05 | 3538 | 50.1 | 4154 | 50.05 | 4129 | 50 | 4156 | 50 | 5427 | 50 | 5607 | 50.05 | 5410 | 50 | 5168 | 50 | 5132 | 50.05 | 5192 | 50 |
| 723.9778 | 12.0663 | 3637 | 50 | 3658 | 50 | 3553 | 50.1 | 4181 | 50 | 4146 | 49.95 | 4190 | 50 | 5449 | 50 | 5627 | 50 | 5426 | 50 | 5191 | 50.05 | 5154 | 50.05 | 5212 | 50.05 |
| 727.9778 | 12.1330 | 3655 | 50.05 | 3680 | 49.9 | 3572 | 50 | 4195 | 50 | 4168 | 50 | 4215 | 50 | 5473 | 50 | 5651 | 49.95 | 5456 | 50 | 5211 | 50.05 | 5178 | 50.05 | 5232 | 50.05 |
| 731.9778 | 12.1996 | 3675 | 50 | 3703 | 49.95 | 3597 | 50 | 4216 | 50 | 4192 | 50 | 4233 | 50 | 5500 | 50 | 5673 | 49.95 | 5468 | 50 | 5234 | 50 | 5200 | 50 | 5258 | 49.95 |
| 735.9778 | 12.2663 | 3692 | 50 | 3723 | 50 | 3607 | 49.95 | 4236 | 50 | 4206 | 50 | 4252 | 50 | 5518 | 50 | 5695 | 50 | 5489 | 50.05 | 5268 | 49.95 | 5216 | 50 | 5280 | 49.9 |
| 739.9778 | 12.3330 | 3714 | 49.95 | 3738 | 50 | 3626 | 50 | 4258 | 50 | 4226 | 50.05 | 4273 | 49.95 | 5533 | 50 | 5729 | 50 | 5502 | 50.05 | 5293 | 49.95 | 5232 | 49.95 | 5297 | 49.95 |
| 743.9778 | 12.3996 | 3720 | 49.95 | 3751 | 50 | 3644 | 50 | 4285 | 50 | 4248 | 50 | 4293 | 50 | 5547 | 50 | 5744 | 50 | 5522 | 49.95 | 5314 | 50 | 5251 | 50 | 5319 | 50 |
| 747.9778 | 12.4663 | 3738 | 49.95 | 3764 | 50.05 | 3656 | 50.05 | 4307 | 50 | 4274 | 49.95 | 4310 | 50 | 5568 | 50 | 5776 | 50 | 5536 | 49.9 | 5327 | 50.05 | 5264 | 50 | 5334 | 50.05 |
| 751.9778 | 12.5330 | 3757 | 50 | 3777 | 50 | 3670 | 50 | 4319 | 49.95 | 4287 | 49.95 | 4329 | 50 | 5573 | 50 | 5799 | 50 | 5549 | 49.9 | 5342 | 50 | 5287 | 50 | 5344 | 49.95 |
| 547.9778 | 9.1330 | 2086 | 73.5 | 2047 | 73.55 | 2041 | 73.55 | 2322 | 73.45 | 2260 | 73.55 | 2340 | 73.6 | 3033 | 73.55 | 3074 | 73.55 | 2997 | 73.55 | 3020 | 73.65 | 3011 | 73.4 | 3080 | 73.6 |
| 551.9778 | 9.1996 | 2101 | 72.75 | 2058 | 72.7 | 2054 | 72.7 | 2340 | 72.75 | 2283 | 72.75 | 2360 | 72.8 | 3070 | 72.7 | 3104 | 72.8 | 3028 | 72.7 | 3045 | 72.75 | 3036 | 72.8 | 3104 | 72.75 |
| 555.9778 | 9.2663 | 2121 | 71.85 | 2072 | 72 | 2074 | 72 | 2364 | 72 | 2306 | 72.05 | 2379 | 71.85 | 3097 | 72 | 3146 | 71.95 | 3051 | 71.95 | 3080 | 71.9 | 3069 | 71.9 | 3133 | 72.1 |
| 559.9778 | 9.3330 | 2136 | 71.25 | 2094 | 71.25 | 2105 | 71.15 | 2386 | 71.25 | 2333 | 71.1 | 2402 | 71.2 | 3140 | 71.25 | 3186 | 71.2 | 3099 | 71.2 | 3112 | 71.3 | 3099 | 71.25 | 3161 | 71.15 |
| 563.9778 | 9.3996 | 2153 | 70.45 | 2116 | 70.3 | 2115 | 70.25 | 2415 | 70.35 | 2357 | 70.35 | 2429 | 70.35 | 3173 | 70.35 | 3225 | 70.35 | 3134 | 70.2 | 3147 | 70.3 | 3131 | 70.45 | 3187 | 70.35 |
| 567.9778 | 9.4663 | 2169 | 69.55 | 2135 | 69.7 | 2123 | 69.65 | 2441 | 69.6 | 2386 | 69.65 | 2459 | 69.6 | 3209 | 69.7 | 3272 | 69.55 | 3169 | 69.6 | 3184 | 69.55 | 3171 | 69.55 | 3230 | 69.65 |
| 571.9778 | 9.5330 | 2199 | 68.9 | 2162 | 68.75 | 2149 | 68.75 | 2471 | 68.8 | 2421 | 68.75 | 2489 | 68.9 | 3257 | 68.8 | 3323 | 68.95 | 3216 | 68.8 | 3222 | 68.85 | 3206 | 68.95 | 3266 | 68.75 |
| 575.9778 | 9.5996 | 2222 | 68.05 | 2189 | 68 | 2177 | 68.05 | 2502 | 68.05 | 2450 | 68 | 2520 | 67.95 | 3303 | 68.1 | 3383 | 68.1 | 3275 | 67.95 | 3267 | 68.05 | 3255 | 68 | 3302 | 68 |
| 579.9778 | 9.6663 | 2252 | 67.3 | 2225 | 67.3 | 2208 | 67.35 | 2538 | 67.3 | 2490 | 67.35 | 2555 | 67.3 | 3372 | 67.3 | 3445 | 67.3 | 3341 | 67.35 | 3316 | 67.3 | 3297 | 67.25 | 3350 | 67.3 |
| 583.9778 | 9.7330 | 2284 | 66.55 | 2260 | 66.5 | 2239 | 66.5 | 2576 | 66.5 | 2530 | 66.6 | 2591 | 66.55 | 3425 | 66.5 | 3516 | 66.6 | 3401 | 66.5 | 3371 | 66.4 | 3360 | 66.65 | 3401 | 66.5 |
| 587.9778 | 9.7996 | 2323 | 65.65 | 2294 | 65.85 | 2297 | 65.85 | 2618 | 65.7 | 2573 | 65.9 | 2638 | 65.7 | 3488 | 65.8 | 3587 | 65.8 | 3470 | 65.8 | 3431 | 65.7 | 3418 | 65.75 | 3456 | 65.9 |
| 591.9778 | 9.8663 | 2358 | 64.85 | 2333 | 64.95 | 2313 | 65 | 2669 | 64.95 | 2625 | 64.9 | 2677 | 65 | 3559 | 64.9 | 3665 | 64.9 | 3538 | 64.95 | 3501 | 64.95 | 3485 | 65.05 | 3516 | 64.95 |
| 595.9778 | 9.9330 | 2406 | 64.15 | 2378 | 64.15 | 2353 | 64.05 | 2713 | 64 | 2672 | 64.15 | 2728 | 64.15 | 3638 | 64.05 | 3745 | 64.15 | 3617 | 64.1 | 3563 | 64.15 | 3553 | 64.15 | 3583 | 64.15 |
| 599.9778 | 9.9996 | 2447 | 63.25 | 2419 | 63.5 | 2398 | 63.4 | 2760 | 63.35 | 2718 | 63.4 | 2775 | 63.45 | 3704 | 63.4 | 3826 | 63.35 | 3686 | 63.45 | 3631 | 63.35 | 3623 | 63.3 | 3649 | 63.45 |
| 603.9778 | 10.0663 | 2486 | 62.7 | 2465 | 62.55 | 2435 | 62.5 | 2812 | 62.65 | 2772 | 62.6 | 2824 | 62.6 | 3784 | 62.55 | 3907 | 62.65 | 3765 | 62.6 | 3707 | 62.65 | 3697 | 62.7 | 3719 | 62.65 |
| 607.9778 | 10.1330 | 2527 | 61.8 | 2503 | 61.8 | 2480 | 61.85 | 2861 | 61.95 | 2823 | 61.75 | 2874 | 61.75 | 3853 | 61.9 | 3988 | 61.8 | 3838 | 61.7 | 3778 | 61.9 | 3769 | 61.75 | 3793 | 61.75 |
| 611.9778 | 10.1996 | 2568 | 61.1 | 2553 | 61.05 | 2519 | 61.1 | 2911 | 61.1 | 2882 | 61.05 | 2925 | 61.1 | 3928 | 61.1 | 4065 | 60.95 | 3917 | 61.1 | 3852 | 61.15 | 3840 | 61 | 3858 | 61.05 |
| 615.9778 | 10.2663 | 2613 | 60.3 | 2595 | 60.25 | 2567 | 60.25 | 2962 | 60.3 | 2934 | 60.3 | 2978 | 60.2 | 4003 | 60.25 | 4145 | 60.3 | 3994 | 60.2 | 3927 | 60.2 | 3907 | 60.4 | 3925 | 60.3 |

| **S3A: PASTING PROPERTIES RAW DATA PART 1 …continuation** | | | | | | | | | | | | | | | | | | | | | | | | | |
| --- | --- | --- | --- | --- | --- | --- | --- | --- | --- | --- | --- | --- | --- | --- | --- | --- | --- | --- | --- | --- | --- | --- | --- | --- | --- |
| **2019-15** | | **BROWN_UNF T1** | | **BROWN_UNF T2** | | **BROWN_UNF T3** | | **BROWN_FORT 1** | | **BROWN_FORT 2** | | **BROWN_FORT 3** | | **MILLED_UNF 1** | | **MILLED_UNF 2** | | **MILLED_UNF 3** | | **MILLED_FORT 1** | | **MILLED_FORT 2** | | **MILLED_FORT 3** | |
| time (sec) | time (min) | visc (cP) | temp (°C) | visc (cP) | temp (°C) | visc (cP) | temp (°C) | visc (cP) | temp (°C) | visc (cP) | temp (°C) | visc (cP) | temp (°C) | visc (cP) | temp (°C) | visc (cP) | temp (°C) | visc (cP) | temp (°C) | visc (cP) | temp (°C) | visc (cP) | temp (°C) | visc (cP) | temp (°C) |
| 619.9778 | 10.3330 | 2659 | 59.3 | 2648 | 59.5 | 2611 | 59.55 | 3010 | 59.45 | 2985 | 59.6 | 3028 | 59.35 | 4080 | 59.6 | 4223 | 59.5 | 4068 | 59.55 | 3997 | 59.4 | 3981 | 59.4 | 3996 | 59.5 |
| 623.9778 | 10.3996 | 2707 | 58.7 | 2694 | 58.7 | 2649 | 58.7 | 3063 | 58.7 | 3036 | 58.7 | 3079 | 58.75 | 4153 | 58.65 | 4302 | 58.55 | 4150 | 58.75 | 4069 | 58.7 | 4048 | 58.75 | 4060 | 58.65 |
| 627.9778 | 10.4663 | 2748 | 57.85 | 2737 | 57.95 | 2693 | 57.9 | 3117 | 57.75 | 3091 | 57.9 | 3133 | 57.85 | 4222 | 57.85 | 4378 | 57.9 | 4214 | 57.8 | 4136 | 57.9 | 4117 | 57.9 | 4131 | 57.95 |
| 631.9778 | 10.5330 | 2792 | 57.1 | 2784 | 57.2 | 2743 | 57.2 | 3165 | 57.15 | 3146 | 57.15 | 3188 | 57.2 | 4293 | 57.1 | 4453 | 57.15 | 4290 | 57.2 | 4201 | 57 | 4181 | 57.1 | 4195 | 57.2 |
| 635.9778 | 10.5996 | 2838 | 56.45 | 2825 | 56.4 | 2792 | 56.3 | 3218 | 56.3 | 3194 | 56.45 | 3238 | 56.45 | 4361 | 56.3 | 4526 | 56.45 | 4361 | 56.35 | 4267 | 56.4 | 4252 | 56.45 | 4258 | 56.45 |
| 639.9778 | 10.6663 | 2881 | 55.5 | 2877 | 55.55 | 2830 | 55.55 | 3267 | 55.65 | 3243 | 55.5 | 3294 | 55.5 | 4434 | 55.7 | 4597 | 55.65 | 4433 | 55.5 | 4330 | 55.6 | 4312 | 55.55 | 4319 | 55.5 |
| 643.9778 | 10.7330 | 2927 | 54.75 | 2919 | 54.8 | 2875 | 54.85 | 3319 | 54.85 | 3291 | 54.8 | 3347 | 54.9 | 4504 | 54.85 | 4673 | 54.8 | 4497 | 54.8 | 4387 | 55 | 4370 | 54.75 | 4382 | 54.85 |
| 647.9778 | 10.7996 | 2975 | 54 | 2962 | 53.95 | 2913 | 54 | 3372 | 53.9 | 3339 | 53.95 | 3395 | 53.95 | 4568 | 54 | 4739 | 53.95 | 4567 | 53.95 | 4448 | 54.05 | 4438 | 54 | 4439 | 54 |
| 651.9778 | 10.8663 | 3012 | 53.2 | 3010 | 53.3 | 2950 | 53.3 | 3419 | 53.25 | 3394 | 53.3 | 3445 | 53.2 | 4637 | 53.25 | 4808 | 53.25 | 4638 | 53.25 | 4509 | 53.2 | 4495 | 53.2 | 4503 | 53.25 |
| 655.9778 | 10.9330 | 3058 | 52.6 | 3052 | 52.4 | 2986 | 52.4 | 3472 | 52.5 | 3451 | 52.45 | 3495 | 52.5 | 4699 | 52.5 | 4881 | 52.35 | 4705 | 52.45 | 4568 | 52.5 | 4552 | 52.6 | 4556 | 52.5 |
| 659.9778 | 10.9996 | 3108 | 51.7 | 3096 | 51.7 | 3030 | 51.7 | 3528 | 51.6 | 3506 | 51.65 | 3548 | 51.65 | 4767 | 51.65 | 4942 | 51.75 | 4763 | 51.7 | 4626 | 51.75 | 4601 | 51.65 | 4611 | 51.6 |
| 663.9778 | 11.0663 | 3144 | 50.85 | 3138 | 50.95 | 3080 | 50.9 | 3582 | 50.9 | 3556 | 50.9 | 3600 | 50.95 | 4826 | 50.9 | 5011 | 50.85 | 4820 | 51 | 4678 | 50.85 | 4650 | 50.85 | 4662 | 50.9 |
| 667.9778 | 11.1330 | 3192 | 50.2 | 3180 | 50.15 | 3121 | 50.1 | 3632 | 50.15 | 3614 | 50.15 | 3648 | 50.2 | 4884 | 50.1 | 5077 | 50.25 | 4887 | 50.2 | 4729 | 50.1 | 4696 | 50.2 | 4717 | 50.15 |
| 671.9778 | 11.1996 | 3231 | 50.1 | 3229 | 50 | 3161 | 49.95 | 3689 | 50.1 | 3660 | 50.05 | 3696 | 50.1 | 4943 | 49.95 | 5138 | 50 | 4944 | 50.05 | 4783 | 49.95 | 4746 | 50.15 | 4763 | 50.15 |
| 675.9778 | 11.2663 | 3267 | 50.1 | 3288 | 50.1 | 3208 | 50.1 | 3741 | 50.1 | 3708 | 50.2 | 3746 | 50.1 | 4997 | 50.15 | 5195 | 50.15 | 5003 | 50.05 | 4828 | 50.1 | 4787 | 50.15 | 4815 | 50.15 |
| 679.9778 | 11.3330 | 3307 | 50.1 | 3323 | 50.2 | 3253 | 50.25 | 3786 | 50.2 | 3752 | 50.2 | 3793 | 50.2 | 5045 | 50.25 | 5249 | 50.2 | 5055 | 50.15 | 4867 | 50.2 | 4830 | 50.15 | 4853 | 50.2 |
| 683.9778 | 11.3996 | 3346 | 50.15 | 3363 | 50.15 | 3280 | 50.2 | 3831 | 50 | 3797 | 50 | 3845 | 50.15 | 5091 | 50.2 | 5297 | 50.1 | 5110 | 50.15 | 4907 | 50.15 | 4865 | 50.05 | 4897 | 50.1 |
| 687.9778 | 11.4663 | 3384 | 50.05 | 3396 | 50.05 | 3312 | 50.05 | 3869 | 50.05 | 3837 | 50.05 | 3888 | 50.1 | 5129 | 50 | 5341 | 50 | 5154 | 50.15 | 4938 | 50.05 | 4897 | 50.05 | 4937 | 50.1 |
| 691.9778 | 11.5330 | 3412 | 50 | 3434 | 50 | 3346 | 50 | 3909 | 50.05 | 3877 | 50.1 | 3923 | 50 | 5167 | 49.95 | 5379 | 50 | 5194 | 50.15 | 4977 | 50.05 | 4931 | 50.05 | 4979 | 50.05 |
| 695.9778 | 11.5996 | 3450 | 50 | 3467 | 50 | 3382 | 50.05 | 3944 | 50 | 3919 | 49.95 | 3968 | 49.9 | 5210 | 50 | 5420 | 50.1 | 5233 | 50 | 5001 | 50.05 | 4969 | 50 | 5009 | 50.05 |
| 699.9778 | 11.6663 | 3482 | 50 | 3500 | 50.1 | 3419 | 50.1 | 3977 | 50.05 | 3948 | 50 | 4007 | 50 | 5248 | 50.1 | 5458 | 50.05 | 5263 | 50 | 5035 | 50.1 | 5000 | 50 | 5045 | 50.05 |
| 703.9778 | 11.7330 | 3513 | 50.1 | 3520 | 50.05 | 3439 | 50 | 4012 | 50.05 | 3985 | 50 | 4037 | 50.1 | 5283 | 50.05 | 5489 | 50 | 5296 | 50 | 5068 | 50.1 | 5024 | 50 | 5070 | 50.1 |
| 707.9778 | 11.7996 | 3538 | 50.1 | 3557 | 49.95 | 3459 | 49.95 | 4041 | 50.05 | 4035 | 50.05 | 4073 | 50.05 | 5321 | 50.05 | 5519 | 49.9 | 5327 | 50.05 | 5094 | 50.05 | 5054 | 50 | 5101 | 50.05 |
| 711.9778 | 11.8663 | 3565 | 50 | 3571 | 50 | 3486 | 50 | 4079 | 50 | 4081 | 50.15 | 4097 | 50.05 | 5366 | 49.95 | 5551 | 49.95 | 5358 | 50.05 | 5119 | 49.95 | 5078 | 49.95 | 5141 | 49.95 |
| 715.9778 | 11.9330 | 3589 | 49.9 | 3604 | 50.05 | 3511 | 50 | 4132 | 50.05 | 4102 | 50.05 | 4128 | 49.95 | 5401 | 49.95 | 5579 | 50.05 | 5380 | 50 | 5147 | 50 | 5102 | 50 | 5165 | 49.95 |
| 719.9778 | 11.9996 | 3610 | 49.9 | 3628 | 50.05 | 3538 | 50.1 | 4154 | 50.05 | 4129 | 50 | 4156 | 50 | 5427 | 50 | 5607 | 50.05 | 5410 | 50 | 5168 | 50 | 5132 | 50.05 | 5192 | 50 |
| 723.9778 | 12.0663 | 3637 | 50 | 3658 | 50 | 3553 | 50.1 | 4181 | 50 | 4146 | 49.95 | 4190 | 50 | 5449 | 50 | 5627 | 50 | 5426 | 50 | 5191 | 50.05 | 5154 | 50.05 | 5212 | 50.05 |
| 727.9778 | 12.1330 | 3655 | 50.05 | 3680 | 49.9 | 3572 | 50 | 4195 | 50 | 4168 | 50 | 4215 | 50 | 5473 | 50 | 5651 | 49.95 | 5456 | 50 | 5211 | 50.05 | 5178 | 50.05 | 5232 | 50.05 |
| 731.9778 | 12.1996 | 3675 | 50 | 3703 | 49.95 | 3597 | 50 | 4216 | 50 | 4192 | 50 | 4233 | 50 | 5500 | 50 | 5673 | 49.95 | 5468 | 50 | 5234 | 50 | 5200 | 50 | 5258 | 49.95 |
| 735.9778 | 12.2663 | 3692 | 50 | 3723 | 50 | 3607 | 49.95 | 4236 | 50 | 4206 | 50 | 4252 | 50 | 5518 | 50 | 5695 | 50 | 5489 | 50.05 | 5268 | 49.95 | 5216 | 50 | 5280 | 49.9 |
| 739.9778 | 12.3330 | 3714 | 49.95 | 3738 | 50 | 3626 | 50 | 4258 | 50 | 4226 | 50.05 | 4273 | 49.95 | 5533 | 50 | 5729 | 50 | 5502 | 50.05 | 5293 | 49.95 | 5232 | 49.95 | 5297 | 49.95 |
| 743.9778 | 12.3996 | 3720 | 49.95 | 3751 | 50 | 3644 | 50 | 4285 | 50 | 4248 | 50 | 4293 | 50 | 5547 | 50 | 5744 | 50 | 5522 | 49.95 | 5314 | 50 | 5251 | 50 | 5319 | 50 |
| 747.9778 | 12.4663 | 3738 | 49.95 | 3764 | 50.05 | 3656 | 50.05 | 4307 | 50 | 4274 | 49.95 | 4310 | 50 | 5568 | 50 | 5776 | 50 | 5536 | 49.9 | 5327 | 50.05 | 5264 | 50 | 5334 | 50.05 |
| 751.9778 | 12.5330 | 3757 | 50 | 3777 | 50 | 3670 | 50 | 4319 | 49.95 | 4287 | 49.95 | 4329 | 50 | 5573 | 50 | 5799 | 50 | 5549 | 49.9 | 5342 | 50 | 5287 | 50 | 5344 | 49.95 |

**Table S3B** Raw data for the pasting properties (Part 2), from which Table 3 was derived

| **S3B: PASTING PROPERTIES RAW DATA PART 2** | | | | | | | | | | | |
| --- | --- | --- | --- | --- | --- | --- | --- | --- | --- | --- | --- |
| Rapid Visco Analyser (Viscosity Profile) Raw Data | | | | | | | | | | | |
| **ENTNO** | **GID** | **Designation** | **Barcode Text** | **Peak_1** | **Trough_1** | **Breakdown** | **Final_**  **Visc** | **Setback** | **Peak_**  **Time** | **Pasting_**  **Temp** | **Retro** |
| 1 | 4249903 | brown_unf_T1 | 201915-00001-0004249903 | 2301 | 2030 | 271 | 3757 | 1456 | 6.13 | 82.3 | 1727 |
| 2 | 4249903 | brown_unf_T2 | 201915-00002-0004249903 | 2217 | 1981 | 236 | 3777 | 1560 | 6.2 | 82.3 | 1796 |
| 3 | 4249903 | brown_unf_T3 | 201915-00003-0004249903 | 2187 | 1983 | 204 | 3670 | 1483 | 6.33 | 79.9 | 1687 |
| 4 | 4249903 | brown_fort_T1 | 201915-00004-0004249903 | 2759 | 2248 | 511 | 4319 | 1560 | 6 | 79.9 | 2071 |
| 5 | 4249903 | brown_fort_T2 | 201915-00005-0004249903 | 2653 | 2167 | 486 | 4287 | 1634 | 6 | 79.95 | 2120 |
| 6 | 4249903 | brown_fort_T3 | 201915-00006-0004249903 | 2768 | 2264 | 504 | 4329 | 1561 | 6.07 | 79.85 | 2065 |
| 7 | 4249903 | milled_unf_T1 | 201915-00007-0004249903 | 3804 | 2886 | 918 | 5573 | 1769 | 6.07 | 79.1 | 2687 |
| 8 | 4249903 | milled_unf_T2 | 201915-00008-0004249903 | 3894 | 2880 | 1014 | 5799 | 1905 | 5.87 | 79.2 | 2919 |
| 9 | 4249903 | milled_unf_T3 | 201915-00009-0004249903 | 3736 | 2833 | 903 | 5549 | 1813 | 5.93 | 79.9 | 2716 |
| 10 | 4249903 | milled_fort_T1 | 201915-00010-0004249903 | 3697 | 2874 | 823 | 5342 | 1645 | 6.13 | 79.8 | 2468 |
| 11 | 4249903 | milled_fort_T2 | 201915-00011-0004249903 | 3672 | 2865 | 807 | 5287 | 1615 | 6.13 | 72.8 | 2422 |
| 12 | 4249903 | milled_fort_T3 | 201915-00012-0004249903 | 3760 | 2957 | 803 | 5344 | 1584 | 6.2 | 77.6 | 2387 |

**Table S4** Raw data for the textural profiles, from which Figure 1 was derived

| S4: TEXTURE ANALYSIS RAW DATA | | | | | | | | | | |
| --- | --- | --- | --- | --- | --- | --- | --- | --- | --- | --- |
| **BROWN RICE SAMPLES** | | | | |  | **MILLED RICE SAMPLES** | | | | |
|  | Hardness  (N) | Adhesiveness (J) | Springiness (mm) | Cohesiveness  (no unit) |  |  | Hardness  (N) | Adhesiveness  (J) | Springiness  (mm) | Cohesiveness  (no unit) |
| **RAW** | 3123.923 | -11.776 | 0.274 | 0.395 |  | **RAW** | 2065.168 | -23.587 | 0.074 | 0.417 |
|  | 3901.212 | -4.275 | 0.171 | 0.456 |  |  | 2280.589 | -16.795 | 0.063 | 0.439 |
|  | 3644.249 | -6.158 | 0.114 | 0.432 |  |  | 2216.324 | -19.609 | 0.073 | 0.4 |
| ave | 3556.46 | -7.40 | 0.19 | 0.43 |  | ave | 2187.36 | -20.00 | 0.07 | 0.42 |
| sd | 396.01 | 3.90 | 0.08 | 0.03 |  | sd | 110.59 | 3.41 | 0.01 | 0.02 |
| **CONTROL** | 2322.497 | -11.057 | 0.108 | 0.45 |  | **CONTROL** | 2030.484 | -13.452 | 0.101 | 0.408 |
|  | 2295.348 | -12.522 | 0.152 | 0.41 |  |  | 2073.7 | -19.319 | 0.105 | 0.361 |
|  | 2296.457 | -11.575 | 0.147 | 0.367 |  |  | 2146.646 | -33.188 | 0.075 | 0.378 |
| ave | 2304.77 | -11.72 | 0.14 | 0.41 |  | ave | 2083.61 | -21.99 | 0.09 | 0.38 |
| sd | 15.36 | 0.74 | 0.02 | 0.04 |  | sd | 58.71 | 10.13 | 0.02 | 0.02 |
| **ONE POT** | 2067.859 | -13.117 | 0.098 | 0.436 |  | **ONE POT** | 1995.44 | -33.795 | 0.106 | 0.478 |
|  | 2078.924 | -6.984 | 0.087 | 0.449 |  |  | 2000.25 | -32.655 | 0.102 | 0.454 |
|  | 2068.591 | -11.757 | 0.163 | 0.433 |  |  | 2020.35 | -28.065 | 0.094 | 0.438 |
| ave | 2071.79 | -10.62 | 0.12 | 0.44 |  | ave | 2005.35 | -31.51 | 0.10 | 0.46 |
| sd | 6.19 | 3.22 | 0.04 | 0.01 |  | sd | 13.21 | 3.03 | 0.01 | 0.02 |
| **STEPWISE** | 2141.262 | -20.738 | 0.107 | 0.409 |  | **STEPWISE** | 1749.469 | -36.007 | 0.132 | 0.359 |
|  | 2103.966 | -15.577 | 0.069 | 0.503 |  |  | 1868.088 | -31.781 | 0.12 | 0.415 |
|  | 1958.876 | -9.441 | 0.07 | 0.418 |  |  | 1688.376 | -42.792 | 0.122 | 0.337 |
| ave | 2068.03 | -15.25 | 0.08 | 0.44 |  | ave | 1768.64 | -36.86 | 0.12 | 0.37 |
| sd | 96.36 | 5.66 | 0.02 | 0.05 |  | sd | 91.38 | 5.55 | 0.01 | 0.04 |
